# Phase separation of YAP-MAML2 differentially regulates the transcriptome

**DOI:** 10.1073/pnas.2310430121

**Published:** 2024-02-05

**Authors:** Chan-I. Chung, Junjiao Yang, Xiaoyu Yang, Hongjiang Liu, Zhimin Ma, Frank Szulzewsky, Eric C. Holland, Yin Shen, Xiaokun Shu

**Affiliations:** ^a^Department of Pharmaceutical Chemistry, University of California–San Francisco, San Francisco, CA 94158; ^b^Cardiovascular Research Institute, University of California–San Francisco, San Francisco, CA 94158; ^c^Department of Neurology, Institute for Human Genetics, Weill Institute for Neurosciences, University of California, San Francisco, CA 94158; ^d^Human Biology Division, Fred Hutchinson Cancer Center, Seattle, WA 98109; ^e^Seattle Tumor Translational Research Center, Fred Hutchinson Cancer Center, Seattle, WA 98109

## Abstract

Phase separation (PS) drives the formation of biomolecular condensates that are emerging biological structures involved in diverse cellular processes. Recent studies have unveiled PS-induced formation of several transcriptional factor (TF) condensates that are transcriptionally active, but how strongly PS promotes gene activation remains unclear. Here, we show that the oncogenic TF fusion Yes-associated protein 1-Mastermind like transcriptional coactivator 2 (YAP-MAML2) undergoes PS and forms liquid-like condensates that bear the hallmarks of transcriptional activity. Furthermore, we examined the contribution of PS to YAP-MAML2-mediated gene expression by developing a chemogenetic tool that dissolves TF condensates, allowing us to compare phase-separated and non-phase-separated conditions at identical YAP-MAML2 protein levels. We found that a small fraction of YAP-MAML2-regulated genes is further affected by PS, which include the canonical YAP target genes *CTGF* and *CYR61*, and other oncogenes. On the other hand, majority of YAP-MAML2-regulated genes are not affected by PS, highlighting that transcription can be activated effectively by diffuse complexes of TFs with the transcriptional machinery. Our work opens new directions in understanding the role of PS in selective modulation of gene expression, suggesting differential roles of PS in biological processes.

## Significance

Recent studies have discovered that phase separation (PS)–induced transcription factor (TF) condensates are transcriptionally active, but how strongly PS promotes gene activation remains unclear. Our work represents an important step in clarifying key questions in transcription and condensate biology fields: The role of PS in gene transcription. Is it a consequence of chromosomal translocation resulting in TF fusion protein condensation from diffuse TF complexes? Or does PS lead to emergent functions beyond those of small complexes? Our work on oncogenic fusion TF YAP (Yes-associated protein)-MAML2 shows that PS has contributing roles, albeit differential, in modulating transcription. We expect that our findings likely extend to many transcriptional condensates, whether they are formed by wild-type transcription factors or pathogenic forms.

Yes-associated protein 1 (YAP1, also known as YAP) and transcriptional co-activator with PDZ-binding motif (TAZ) are transcriptional coactivators and the principal effectors of the Hippo pathway (1, 2). Aberrant activation of YAP/TAZ is implicated in human cancer (3). Generally, de-regulated YAP/TAZ activity usually occurs through the inactivation of upstream Hippo pathway tumor suppressors (such as NF2/Merlin, LATS1/2, or FAT1-4). Furthermore, several tumor-associated YAP fusions that result from chromosomal translocations have been identified in patient samples (3) and we and others have shown that, among other things, these fusions stabilize oncogenic YAP activity through a constitutive nuclear localization of the fusion protein, mediated through nuclear localization signals in the sequence of the C-terminal fusion partners (4–7). This gene fusion constitutes an alternative route for achieving de-regulated YAP/TAZ activity (3–5). YAP-MAML2 (or YAP1-MAML2) translocation is one of the most common YAP fusions and is most frequently found in NF2-wild-type meningiomas and poroma (7, 8). Furthermore, we have recently shown that YAP-MAML2 activates the expression of several canonical YAP target genes and that exogenous expression of YAP-MAML2 in mice induces the formation of meningioma-like tumors, suggesting that it is an oncogenic driver (4).

Recent studies of several transcriptional factors (TFs) show that they undergo phase separation (PS) to form biomolecular condensates (9–15), including the transcriptional effectors of the Hippo pathway, YAP and TAZ, when the concentration of the proteins surpasses a threshold caused by an environmental stimulus such as osmotic stress for YAP (16). These TF condensates are further shown to be transcriptionally active (17–22). For example, the YAP and TAZ condensates contain transcriptional machinery (16, 23, 24). TF condensates can also form via genetic fusion events caused by chromosomal translocation, such as NUP98-HOXA9 (25). Such fusion genes often retain the intrinsically disordered region (IDR) that promotes PS of the fusion proteins.

Although many TF condensates compartmentalize transcriptional machinery (9–12, 15), whether PS really changes transcriptional output is still under debate (26). Answers to this key question are hampered by conceptual and technical challenges (26). Many studies have employed mutagenesis-based approaches that introduce mutations to change phase behavior in order to correlate the driving force for PS with transcription (11, 16). These mutations are often introduced into the TF’s activation domain that harbors the IDRs mediating PS, but the activation domains often interact with the Mediator that loops the enhancer to promoter via interacting with RNA polymerase II and general transcription factors (27). For example, the activation domain of the transcription factor GCN4 has been characterized to interact with the Mediator (28). Thus, the mutations that are introduced to the activation domain in order to block PS will likely also impact the ability of TFs to form diffuse complexes with the transcriptional machinery (10, 25). Interestingly, recent work shows that roles of IDR in condensation and protein interaction could be separated because they may involve different residues (29). Unfortunately, many previous studies reached conclusions that PS activated transcription, based on the mutagenesis studies that blocking PS by the mutations led to reduced transcriptional output, without key data that these mutations do not affect interaction of the TFs with the Mediator and transcriptional machinery (10, 11, 16, 25). This means that the observed reduction in transcription can be caused by the blocked PS and/or by the reduced interaction with the transcriptional machinery. Therefore, it is still under debate whether the transcriptional output in the presence of TF condensates would also be achieved in the absence of PS. Determining the role of PS in transcription is thus much needed. The conceptual and technical challenges call for new tools that enable us to assess transcriptional activity upon dissolving condensates without introducing mutations or changing expression levels.

Here, we develop a chemogenetic tool, dubbed SPARK-OFF, which we use to dissolve YAP-MAML2 condensates to compare gene expression between the phase-separated YAP-MAML2 that contains condensates and the equi-concentrated YAP-MAML2 without PS after dissolution of the condensates by SPARK-OFF. We find that YAP-MAML2 condensates are transcriptionally active and largely regulate gene expression similar to diffuse YAP-MAML2. However, the expression of a small fraction of genes is altered further by PS, including the canonical YAP target genes *CTGF* and *CYR61*. Our work thus shows a role for PS of the transcription factors in transcription but simultaneously shows that phase-separated TFs do not necessarily mediate all of the enhanced transcriptional output by the TF.

## Results

### YAP-MAML2 Forms Liquid-Like Condensates.

**Fig. 1.**
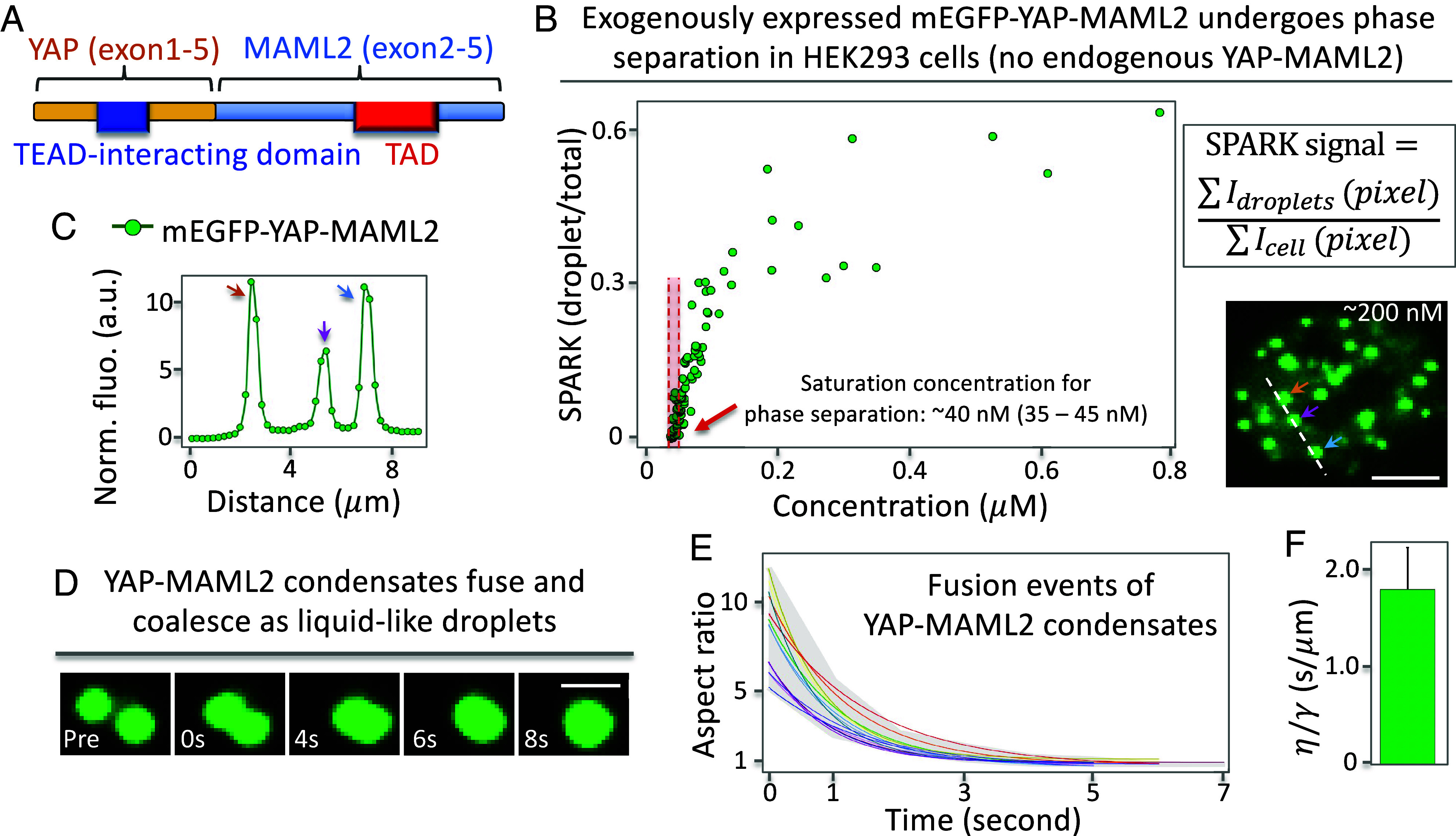
YAP-MAML2 undergoes PS forming liquid-like condensates. (*A*) Schematic of the YAP-MAML2 fusion protein, which is composed of the N-terminal YAP1 (exons 1 to 5) and C-terminal MAML2 (exons 2 to 5). (*B*) Formation of punctate structures of the YAP-MAML2 fusion protein as a function of its concentration. SPARK signal is defined as a ratio of total droplet intensity summarized at all pixels divided by total fluorescence (including droplet and diffuse fluorescence). Arrows point to the condensates. (*C*) Fluorescence intensity profile along the position outlined in the images shown by the dashed line in *B*. (*D*–*F*) Fusion events between YAP-MAML2 condensates. (*D*) Fluorescence images of time course. (*E*) quantitative analysis of the fusion events. Individual lines represent the best-fit line for individual fusion events. Gray shade shows the range of aspect ratio values from all events. (*F*) Mean inverse capillary velocity extracted from fusion events (n = 14). Error bar represents SD. [Scale bars, 5 μm (*B*), 1 μm (*D*).]

The YAP-MAML2 fusion protein retains the TEA Domain Transcription Factor (TEAD)-binding domain of YAP and the transactivation domain of MAML2 (Fig. 1*A*). Here, we examined whether YAP-MAML2 forms puncta and whether they have liquid-like properties. First, we tagged YAP-MAML2 by monomeric enhanced green fluorescent protein (mEGFP) and conducted live-cell imaging. Fluorescence imaging showed that mEGFP-YAP-MAML2 formed punctate structures that are dependent on protein concentration. Quantitative analysis showed that the fusion protein formed punctate structures above a threshold concentration (i.e., saturation concentration): ~40 nM (35 to 45 nM) (Fig. 1*B*). Here, we estimated mEGFP-YAP-MAML2 protein concentration in single cells by comparing mEGFP fluorescence intensity with that of purified mEGFP (*SI Appendix*, Fig. S1). Furthermore, the punctate structures have 5 to 10× higher density than the diffusive state based on the fluorescence intensity (Fig. 1 *B* and *C*). These results suggest that mEGFP-YAP-MAML2 forms condensates via PS (30, 31). We also conducted immunofluorescence (IF) imaging of the ES-2 ovarian carcinoma cells that contain endogenous YAP-MAML2 fusion, which revealed punctate structures with 5 to 10× higher density than dilute phase (*SI Appendix*, Fig. S2 *A* and *B*). We estimated concentration of endogenous YAP-MAML2 to be ~140 nM (*SI Appendix*, Fig. S3 and *Materials and Methods*), which is above the saturation concentration.

Next, we carried out time-lapse imaging and observed that the puncta can fuse and coalesce within a few seconds (Fig. 1*D*). The fusing puncta initially formed a dumbbell shape, which over time relaxed to a spherical shape. Many protein condensates have been characterized to contain viscoelastic material properties due to the architecture of the proteins that are compressible (31–33). Thus, this fusion oncoprotein condensates are most likely viscoelastic. Quantitative analysis showed that aspect ratio of the fusing puncta over time fits well to a single exponential curve (Fig. 1*E*), suggesting that these condensates contain liquid-like properties (34, 35). Lastly, we used the data to determine the inverse capillary velocity (=*η/γ*; γ is surface tension of the droplet; *η* is viscosity), which was 1.8 ± 0.4 (s/µm) (Fig. 1*F*). Here, the dynamic process of fusion is likely much slower than shear relaxation, thus the condensates behave as Newtonian fluid (36). Taken together, our data suggest that mEGFP-YAP-MAML2 undergoes PS forming liquid-like condensates.

### YAP-MAML2 Condensates Contain Transcriptional Machinery and Nascent RNA.

**Fig. 2.**
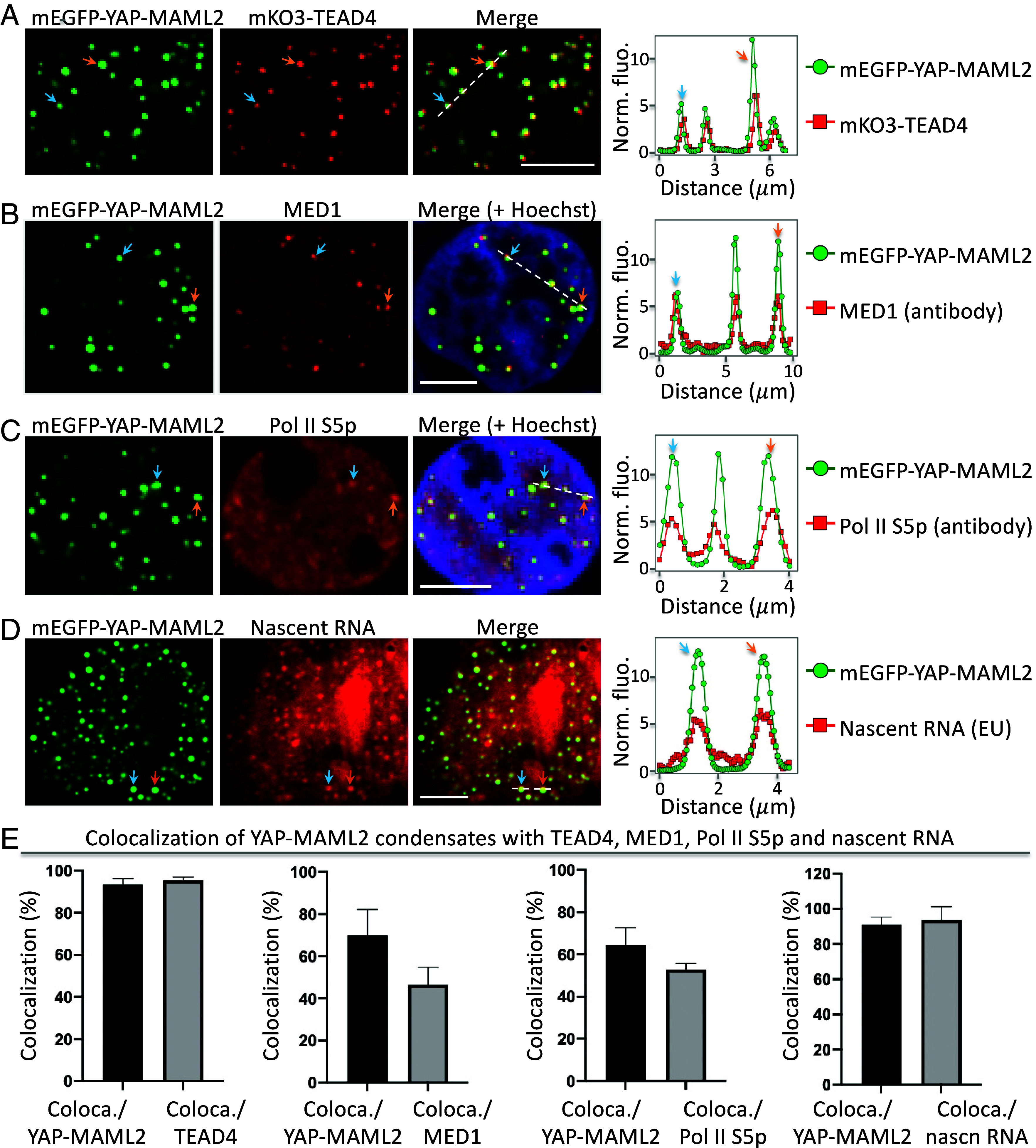
YAP-MAML2 condensates contain transcriptional machinery and nascent RNA. (*A*) Fluorescence images of mEGFP-YAP-MAML2 and MAX-mKO3 in HEK293 cells. The arrows point to representative condensates. The fluorescence intensity profile (*Right*) is extracted from the position shown by the dashed line. Colocalization in example condensates is indicated by arrows. (*B*) Fluorescence images of YAP-MAML2 condensates with IF-imaged MED1. (*C*) Fluorescence images of YAP-MAML2 condensates with IF-imaged Pol II S5p. (*D*) Fluorescence images of YAP-MAML2 condensates with nascent RNA labeled by 5-ethynyluridine. (*E*) Percentage of YAP-MAML2 condensates that colocalize with puncta of other components. The percentage is determined by the ratio of coloca./YAP-MAML2 = number of colocalized condensates between YAP-MAML2 and TEAD4 divided by number of YAP-MAML2 condensates. Equivalent analysis for other pairs is also shown. Data are mean ± SD (n = 13 cells). [Scale bars, 5 μm (*A*–*D*).]

To examine whether the YAP-MAML2 condensates are transcriptionally active, we first asked whether YAP-MAML2 condensates contain the obligatory DNA-binding and dimerization partner TEA Domain Transcription Factor 4 (TEAD4). To visualize TEAD4 in living cells, we labeled it with the red fluorescent protein (FP) mKO3. Multicolor fluorescence imaging showed that YAP-MAML2 condensates contained TEAD4 (Fig. 2*A*), which is consistent with the fact that YAP-MAML2 contains the TEAD-binding domain. Next, IF imaging revealed punctate localization of the Mediator of RNA polymerase II transcription subunit 1 (MED1), consistent with previous studies. Furthermore, YAP-MAML2-positive condensates were a subset of MED1 condensates (Fig. 2*B*), indicating that YAP-MAML2 condensates contain MED1. Third, we stained the cells with antibodies against phosphorylated Pol II at Ser5 (Pol II S5p) at the C-terminal domain. IF imaging showed punctate structures of Pol II S5p, which colocalized with YAP-MAML2 condensates based on two-color imaging (Fig. 2*C*). Therefore, our data indicate that YAP-MAML2 condensates contain the transcriptional machinery.

We next examined whether the YAP-MAML2 condensates contain nascent RNA. We incubated cells with the uridine analog 5-ethynyluridine (EU) for 1 h so that EU was incorporated into newly transcribed RNA. The EU-labeled nascent RNA was detected through a copper (I)-catalyzed cycloaddition reaction (i.e., “click” chemistry) using azides labeled with red fluorescent dyes (37). Fluorescence imaging revealed several punctate structures (Fig. 2*D*), many of which colocalized with the YAP-MAML2 condensates, suggesting that these YAP-MAML2 condensates contain nascent RNAs.

Lastly, we quantified the colocalization of YAP-MAML2 condensates with TEAD4, MED1, Pol II S5p, and nascent RNAs (Fig. 2*E*). We calculated that ~94% of YAP-MAML2 condensates contained TEAD4. The percentage of YAP-MAML2 condensates that contain MED1, Pol II S5p, and nascent RNAs is ~70%, 65%, and 91% respectively. Therefore, our data show that YAP-MAML2 condensates have the hallmarks of transcriptional activity.

### YAP-MAML2 Condensates Are Dynamically Regulated during Cell Mitosis.

Biomolecular condensates have emergent properties that small diffuse complexes do not have, including ripening and coalescence. Coalescence of chromatin-bound transcription factor condensates has the potential to bring distant genomic loci into proximity and concentrate transcriptional machinery and potentially influence transcriptional programs (25). Hence, the lifetimes of the condensates, including the timescales of their assembly and disassembly, are expected to influence their function. Previous studies show that many biomolecular condensates disassemble during mitosis (38). Here, we examined whether YAP-MAML2 condensates were also dynamically regulated during the cell cycle.

**Fig. 3.**
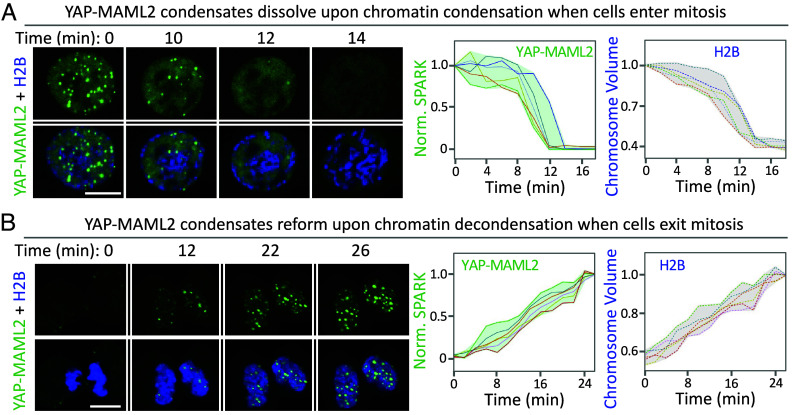
YAP-MAML2 condensates dynamically disassemble and reassemble upon mitotic entry and exit. (*A*) Time-lapse images of HEK293 cells expressing mEGFP-YAP-MAML2 upon mitotic entry. The cells co-expressed monomeric infrared fluorescent protein (mIFP)-tagged H2B (in blue). Chromosome volume was calculated based on mIFP-H2B fluorescence. (*Right*) quantitative analysis of YAP-MAML2 condensate dissolution and chromosome condensation over time. Each line represents single cell traces (n = 6 cells). (*B*) Time-lapse images of HEK293 cells expressing mEGFP-YAP-MAML2 upon mitotic exit. (*Right*) quantitative analysis of YAP-MAML2 condensate reformation and chromosome de-condensation. Each line represents single cell traces (n = 6 cells). (Scale bars, 10 μm.)

Live-cell fluorescence imaging showed that YAP-MAML2 condensates dissolved when cells entered mitosis (Fig. 3 *A*, *Left*). Upon mitotic entry, chromatin condenses and becomes more compacted than that in the interphase. It has been well established that many transcription factors disengage from chromatin when cells enter mitosis (39–41). We thus decided to investigate the relationship between YAP-MAML2 condensate dissolution and chromatin condensation. To visualize the chromatin, we labeled histone 2B (H2B) with a near-infrared FP mIFP (42–46). This allowed us to quantify the volume of chromatin using FP-labeled H2B (47). Time-lapse imaging revealed that YAP-MAML2 condensates dissolved at the same time when the chromatin condenses upon mitotic entry (Fig. 3 *A*, *Right*). YAP-MAML2 condensates dissolved 2 min before nuclear envelope breakdown (Fig. 3*A*, T = 14 min.).

Next, we examined whether the diffuse YAP-MAML2 reformed condensates when cells exit mitosis. Time-lapse imaging revealed that indeed upon mitotic exit, YAP-MAML2 condensates reappeared. We also observed that the chromatin decondensed during mitotic exit, consistent with previous studies (47). The reformation of YAP-MAML2 condensates occurred at the same time as the chromatin de-condensation upon mitotic exit (Fig. 3*B*). Our study thus reveals that YAP-MAML2 condensates are dynamically regulated during mitosis and that disassembly and reassembly of the condensates are correlated with chromatin condensation and de-condensation, respectively.

### A Chemogenetic Tool SPARK-OFF Is Designed to Dissolve Protein Condensates.

**Fig. 4.**
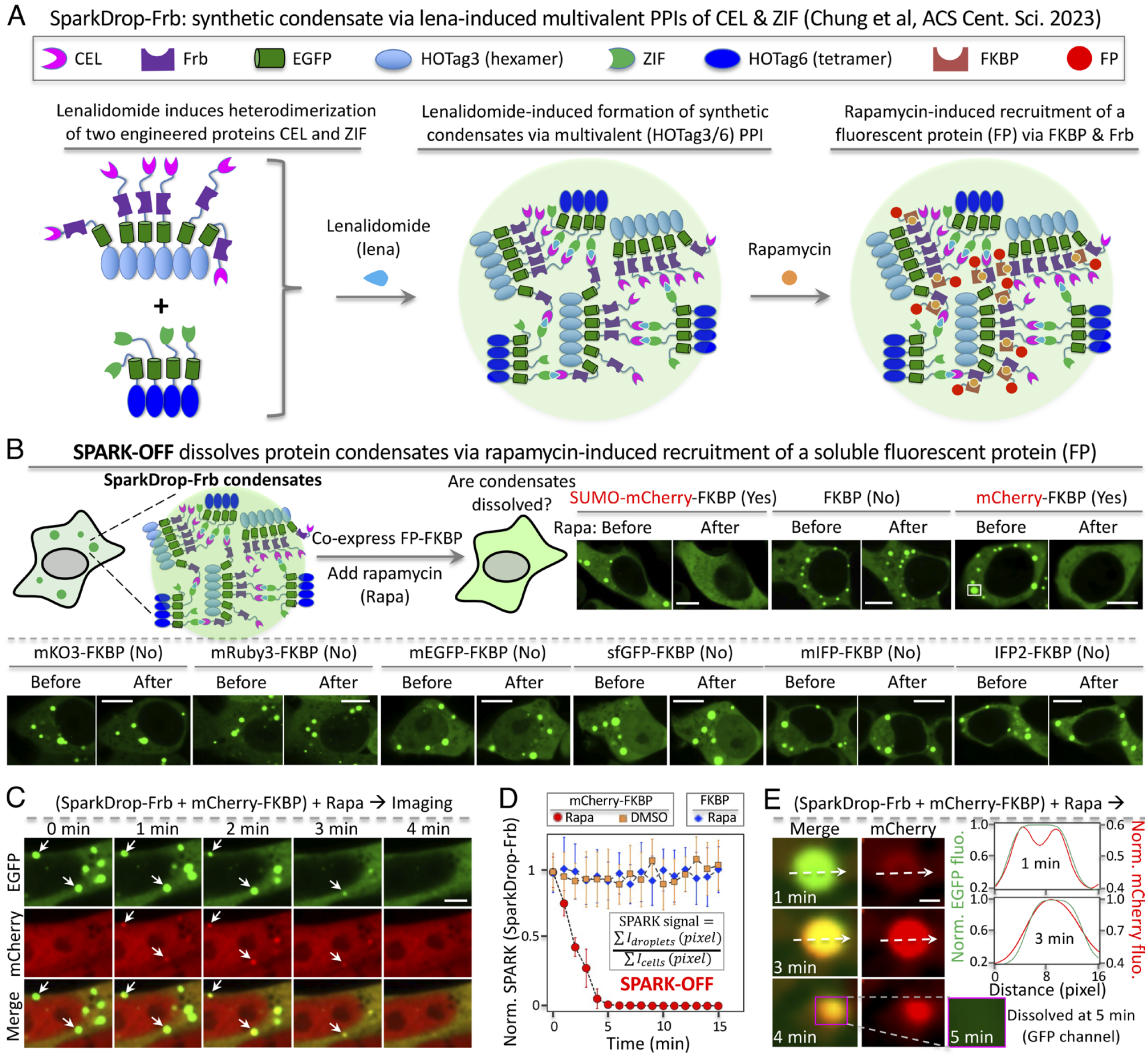
Designing a chemogenetic tool SPARK-OFF that dissolves protein condensates. (*A*) Schematic of synthetic condensates SparkDrop-Frb. (*B*) Schematic of SPARK-OFF and fluorescence images of HEK293 cells. The SparkDrop condensates are GFP-tagged synthetic condensates that form via multivalent interactions. Frb is tagged to the SparkDrop. The cells co-expressed FKBP-fused FPs. The cells were treated with rapamycin to induce the interaction between Frb and FKBP, resulting in recruitment of the FPs to the 20 SparkDrop condensates. SPARK-OFF uses mCherry to dissolve condensates. (*C*) Time-lapse images of HEK293 cells showing dissolution of SparkDrop condensates upon rapamycin-induced recruitment of mCherry. Arrows point to example condensates that are dissolved by SPARK-OFF. (*D*) Quantification of the condensate dissolution over time by rapamycin-activated SPARK-OFF by calculating SPARK signal that is defined by the ratio of the GFP in the condensates over total GFP. The SPARK signal at time 0 is normalized to 1. (n = 3 cells). (*E*) Time-lapse images of HEK293 cells showing mCherry recruitment to SparkDrop condensates and subsequent dissolution of the condensates. (*Right*) the fluorescence intensity profile along the position outlined in the images shown by the dashed lines. [Scale bar: 10 μm (*B*), 5 μm (*C*), 1 μm (*E*).]

Our data show that while the YAP-MAML2 condensates are transcriptionally active, they dissolve during mitosis and thus have a limited lifetime. Consequently, if PS is essential to its transcriptional activity, one would expect a reduction in YAP-MAML2-mediated expression when they dissolve. Therefore, we decided to determine the role of PS of YAP-MAML2 in transcription. To do this, we designed a chemogenetic tool that is able to dissolve condensates without introducing mutations or changing protein abundance levels. To achieve this, we sought to utilize and recruit highly soluble proteins, such as small ubiquitin-like modifier (SUMO), using a small molecule–inducible system. SUMO has been used as a fusion tag in purifying low-solubility proteins (48). Here, we used the molecular glue rapamycin-inducible FK506-binding protein (FKBP) and FKBP-rapamycin binding (Frb) domain hetero-dimer (49). This system has been widely used in investigating cellular processes and signaling by controlling protein locations and interactions, as well as enzyme activities (50–53). To demonstrate this approach, we fused SUMO to FKBP and used the synthetic condensates SparkDrop that forms via multivalent interactions and contains GFP and we incorporated Frb into SparkDrop (SparkDrop-Frb) (Fig. 4*A*) (54). The rationale of using SUMO is that its high solubility may increase interaction between proteins and solvents relative to protein–protein and solvent–solvent interactions, which will increase saturation concentration, leading to condensate dissolution (31). To visualize the dissolution process, we tagged the FKBP fusion with a red FP mCherry (SUMO-mCherry-FKBP). Addition of rapamycin led to dissolution of SparkDrop condensates based on the green fluorescence of GFP, whereas the control construct that only contains FKBP itself without SUMO did not (Fig. 4*B*). Interestingly, another control construct containing mCherry-FKBP, surprisingly dissolved the SparkDrop condensates (Fig. 4*B*). This suggests that mCherry might be able to dissolve protein condensates. We further examined many different FPs, but none of them showed obvious dissolution of SparkDrop condensates (Fig. 4*B* and *SI Appendix*, Fig. S4).

To further characterize the dissolution process via the mCherry-based approach, we conducted time-lapse imaging. Upon addition of rapamycin, red fluorescence appeared at the location of the green SparkDrop condensates (Fig. 4*C*). Then, these condensates quickly dissolved. The dissolution process was completed within 5 min (Fig. 4*D*). In contrast, the SparkDrop condensates alone were stable over time in Dimethyl sulfoxide (DMSO)-incubated cells. Furthermore, rapamycin itself without mCherry did not lead to condensate dissolution. We further characterized the dissolution process with high spatial resolution, which showed that mCherry first partitions into the outer layer of SparkDrop condensates and then diffuses into the core, followed by rapid dissolution of the condensates (Fig. 4*E*). These results suggest that indeed mCherry partitioned into the SparkDrop condensates via rapamycin-inducible dimerization of FKBP and Frb and dissolved the SparkDrop condensates. mCherry is advantageous to SUMO because SUMO is involved in many signaling processes via, for example, SUMO interacting proteins. However, mCherry is a FP cloned from corals and is less likely to perturb signaling processes. mCherry has been widely used as a protein tag in molecular and cell biology, such as for monitoring protein location and trafficking (55, 56). We dubbed this chemogenetic system SPARK-OFF for the dissolution of protein condensates.

### Demonstration of SPARK-OFF in Dissolving Transcriptional Condensates in the Nucleus.

**Fig. 5.**
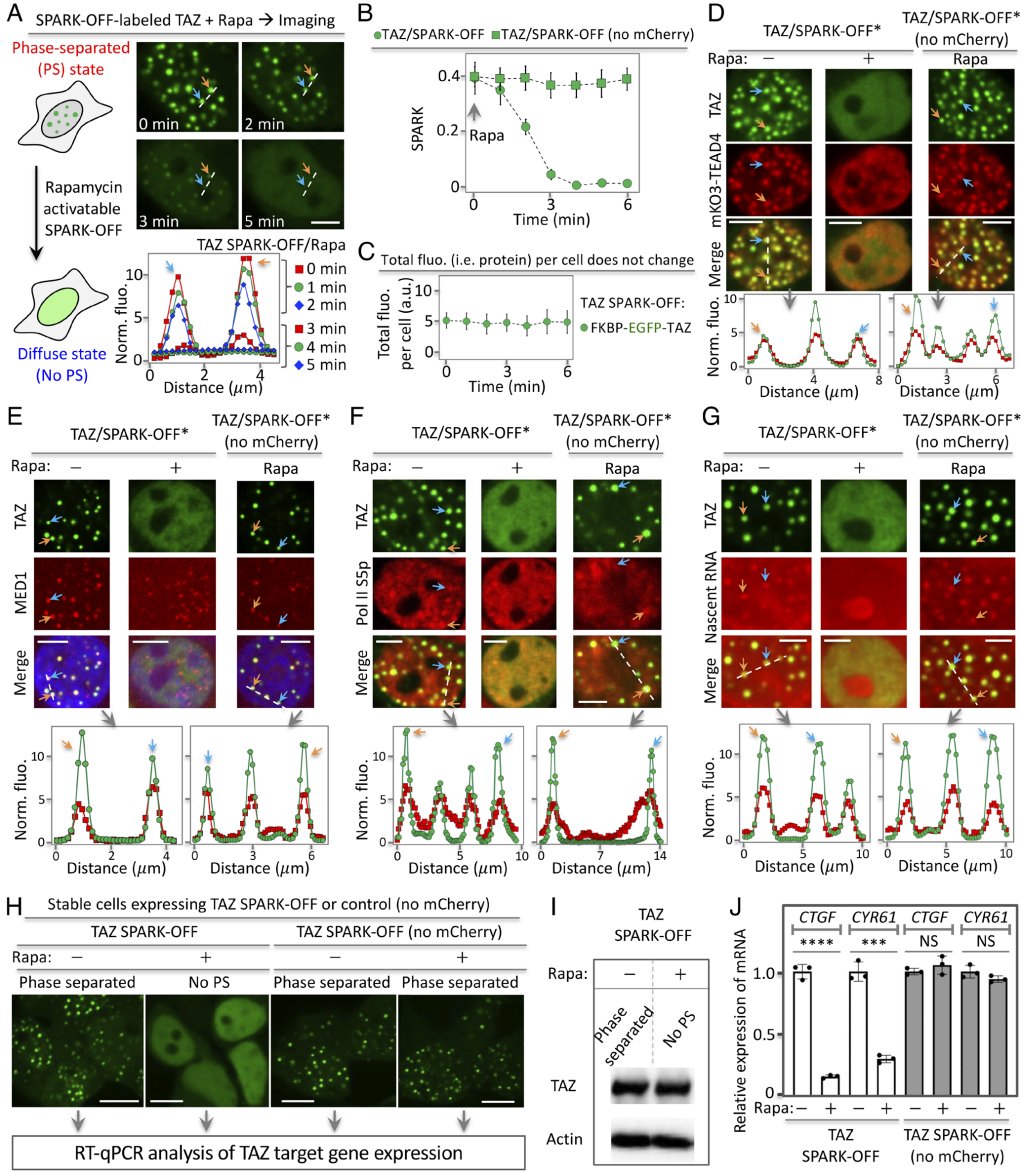
The chemogenetic tool SPARK-OFF reveals the role of TAZ PS on transcription. (*A*) Rapamycin-activatable SPARK-OFF dissolves TAZ condensates without change of protein level. The HEK293 cells expressed FKBP-EGFP-TAZ and NLS-mCherry-Frb. (*B*) Quantitative analysis of SPARK-OFF-driven dissolution of TAZ condensates over time by calculating SPARK signal over time. Data are mean ± SD (n = 3). (*C*) Total fluorescence of TAZ per cell over time upon SPARK-OFF. Data are mean ± SD (n = 3). (*D*–*G*) SPARK-OFF*-driven dissolution of TAZ condensates led to condensate dissolution of the of the DNA-binding and dimerization partner TEAD4 (*D*), transcriptional machinery including MED1 (*E*) and Pol II S5p (*F*), and nascent RNAs (*G*). SPARK-OFF* uses a non-fluorescent mCherry mutant (Y66F). (*H*) Fluorescent images of stable cells expressing SPARK-OFF-tagged TAZ or the control. The cells were treated with rapamycin or DMSO, followed by RT-qPCR analysis. No PS: no phase separation. (*I*) Western blot showing TAZ protein abundance level. (*J*) RT-qPCR analysis of the expression levels of two TAZ target genes in cells without and with PS of TAZ. Data are mean ± SD (n = 3). ****P-value < 0.0001, ***P-value < 0.001. NS, not significant. [Scale bars: 5 μm (*A* and *D*–*G*), 10 μm (*H*).]

To demonstrate the general utility of SPARK-OFF in dissolving condensates of transcription factors, we applied it to TAZ, a normal transcription factor that forms biomolecular condensates (24). First, we tagged SPARK-OFF to TAZ (TAZ/SPARK-OFF). Indeed, TAZ/SPARK-OFF formed condensates in the HEK293 cells (Fig. 5*A*). After activation of SPARK-OFF by rapamycin, these condensates were dissolved within 4 min (Fig. 5*B*). As a control, rapamycin alone did not dissolve the condensates using the TAZ/SPARK-OFF control without mCherry. The total fluorescence per cell did not change, indicating that the total amount of TAZ per cell was constant (Fig. 5*C*). Therefore, SPARK-OFF dissolved TAZ condensates while keeping the total amount of TAZ per cell unchanged.

We next showed that the SPARK-OFF-tagged TAZ condensates contained TEAD4 (Fig. 5*D*). Here, for multicolor imaging, we introduced a single mutation Y72F to mCherry so that it is non-fluorescent. We named this non-fluorescent mCherry-based system as SPARK-OFF*. Our results show that the TAZ condensates contained TEAD4 (*SI Appendix*, Fig. S5). Live-cell imaging showed that dissolution of TAZ condensates led to dissolution of TEAD4 condensates (Fig. 5*D*), suggesting that TEAD4 is recruited to TAZ condensates and that TEAD4 PS is dependent on TAZ condensates.

Lastly, we showed that the SPARK-OFF* tagged TAZ condensates also contained transcriptional machinery including MED1 and Pol II S5p and nascent RNAs. Around 72%, 66%, and 82% of TAZ condensates contained MED1, Pol II S5p, and nascent RNAs, respectively (*SI Appendix*, Fig. S5). Dissolution of TAZ condensates dissolved condensates of transcription machinery, including MED1 (Fig. 5*E*), Pol II-S5p (Fig. 5*F*), and nascent RNAs (Fig. 5*G*). As a control, rapamycin alone did not dissolve these condensates. Therefore, our data indicate that SPARK-OFF is able to dissolve transcription factor condensates while keeping the total protein concentration in the cell unchanged.

### Demonstration of SPARK-OFF in Determining the Role of PS in Transcriptional Regulation.

We further demonstrated that SPARK-OFF could be used to characterize the role of PS of transcription factors. Here, we examined whether the SPARK-OFF-based dissolution of TAZ condensates regulated the expression of the two canonical TAZ target genes *CTGF* and *CYR61*. First, we engineered stable HEK293 cells expressing SPARK-OFF-tagged TAZ. Activation of SPARK-OFF by rapamycin-dissolved TAZ condensates (Fig. 5*H*). Western blot analysis confirmed that TAZ protein levels were unchanged upon SPARK-OFF-induced dissolution of the TAZ condensates (Fig. 5*I*). This enabled us to examine the role of PS on transcription, by comparing the phase-separated TAZ to the equally concentrated TAZ without PS. RT-qPCR analysis showed that mRNA levels of *CTGF* and *CYR61* decreased by 84% and 70%, respectively, upon dissolution of TAZ condensates by SPARK-OFF (Fig. 5*J*). As a control, rapamycin itself did not decrease the mRNA levels, which is consistent with the above data that rapamycin alone did not dissolve TAZ condensates.

Our data thus demonstrated that SPARK-OFF indeed could control the TAZ condensates and that PS of TAZ enhances transcription of the two target genes, consistent with previous studies (24). Therefore, we show that SPARK-OFF is a powerful tool to manipulate transcription factor condensates and to understand the role of PS in transcription.

### Dissolution of YAP-MAML2 Condensates Decreases Transcription of the YAP Target Genes.

**Fig. 6.**
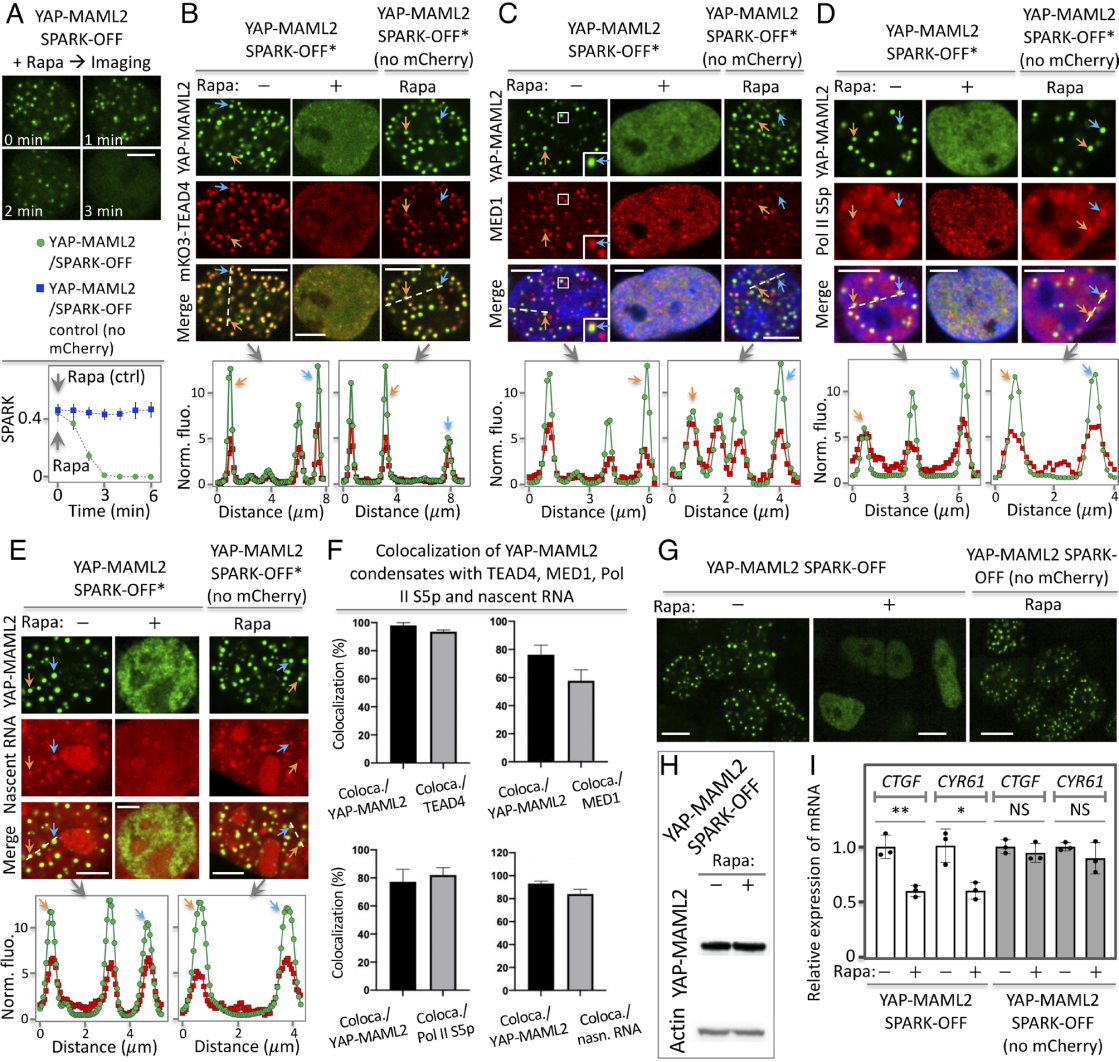
The chemogenetic tool SPARK-OFF reveals role of YAP-MAML2 PS on transcription. (*A*) Rapamycin-activatable SPARK-OFF dissolves YAP-MAML2 condensates. The HEK293 cells expressed FKBP-mEGFP-YAP-MAML2 and NLS-mCherry-Frb. Data are mean ± SD (n = 3). (*B*–*E*) SPARK-OFF*-driven dissolution of YAP-MAML2 condensates led to condensate dissolution of TEAD4 (*B*), transcriptional machinery including MED1 (*C*) and Pol II S5p (*D*), and nascent RNAs (*E*). (*F*) Percentage of YAP-MAML2 condensates that colocalize with puncta of other components. The percentage is determined by the ratio of coloca./YAP-MAML2 = number of colocalized condensates between YAP-MAML2 and TEAD4 divided by number of YAP-MAML2 condensates. Equivalent analysis for other pairs is also shown. Data are mean ± SD (n = 11 cells). (*G*) Fluorescent images of stable cells expressing SPARK-OFF-tagged YAP-MAML2 or the control. The cells were treated with rapamycin or DMSO, followed by RT-qPCR analysis. No PS: no phase separation. (*H*) Western blot showing YAP-MAML2 protein abundance level. (*I*) RT-qPCR analysis of the expression levels of two YAP target genes in cells without and with PS of YAP-MAML2. Data are mean ± SD (n = 3). **P-value < 0.01, *P-value < 0.05. NS, not significant. [Scale bars: 5 μm (*A*–*E*), 10 μm (*G*).]

After successful demonstration of SPARK-OFF in manipulating TAZ condensates, we applied SPARK-OFF to dissolve the YAP-MAML2 condensates. First, we demonstrated that SPARK-OFF labeling did not perturb YAP-MAML2 condensation since the SPARK-OFF-tagged YAP-MAML2 formed condensates in HEK293 cells (Fig. 6*A*). Furthermore, the saturation concentration of the labeled YAP-MAML2 for PS is also ~40 nM (30 to 50 nM), which is similar to that of mEGFP-labeled YAP-MAML2 (Fig. 1*B*). Activation of SPARK-OFF by rapamycin dissolved these condensates within ~3 min. As a control, rapamycin alone did not dissolve the condensates using the YAP-MAML2/SPARK-OFF control without mCherry. The total fluorescence per cell did not change, suggesting that the total amount of YAP-MAML2 per cell was constant (*SI Appendix*, Fig. S6). We further determined that rapamycin shifted saturation concentration of SPARK-OFF-labeled YAP-MAML2 for PS upward from ~40 nM (30 to 50 nM) to ~150 nM (100 to 200 nM) (*SI Appendix*, Fig. S7). While rapamycin dissolved the SPARK-OFF-labeled YAP-MAML2, it had no effect on nuclear condensates such as nucleoli that are not labeled by SPARK-OFF (Movie S1). These results suggest that rapamycin-activatable SPARK-OFF specifically dissolves the condensates that are labeled by the chemogenetic tools by shifting the saturation concentration upward (31, 32), and it has no effect on unlabeled condensates, which is advantageous to and overcomes the problems of 1,6-hexanediol that non-specifically dissolves condensates (57–59).

Second, we demonstrated that the SPARK-OFF-labeling did not perturb transcriptional activity of the YAP-MAML2 condensates, as they contained TEAD4, the transcription machinery including MED1 and Pol II S5p, and nascent RNA (Fig. 6 *B*–*E*). Quantitative analysis showed that ~98% of YAP-MAML2 condensates contained TEAD4 (Fig. 6*F*). Around 76%, 77%, and 93% of YAP-MAML2 condensates contained MED1, Pol II S5p, and nascent RNAs, respectively (Fig. 6*F*).

Third, similar to our results with SPARK-OFF-tagged TAZ condensates, time-lapse imaging showed that the dissolution of YAP-MAML2 condensates led to the dissolution of TEAD4 condensates (Fig. 6*B*), suggesting that TEAD4 was recruited to the YAP-MAML2 condensates and that PS of TEAD4 was dependent on YAP-MAML2 condensates. Dissolution of the YAP-MAML2 condensates also dissolved condensates of the associated transcription machinery including MED1 and Pol II S5p and nascent RNAs (Fig. 6 *C*–*E*).

Lastly, we examined whether dissolution of YAP-MAML2 condensates affects the transcription of YAP-MAML2 target genes. The protein levels of the YAP-MAML2 were unchanged upon dissolution (Fig. 6 *G* and *H* and *SI Appendix*, Fig. S6). RT-qPCR analysis revealed that dissolution of YAP-MAML2 condensates decreased mRNA levels of *CTGF* and *CYR61* by ~ 40% (Fig. 6*I*). By contrast, rapamycin alone did not affect the mRNA levels of these genes, suggesting that rapamycin itself has no effect on the transcription of these genes. Therefore, our data indicate that PS of YAP-MAML2 promotes gene expression of *CTGF* and *CYR61*.

### YAP-MAML2 Condensates Regulate YAP Target Genes.

**Fig. 7.**
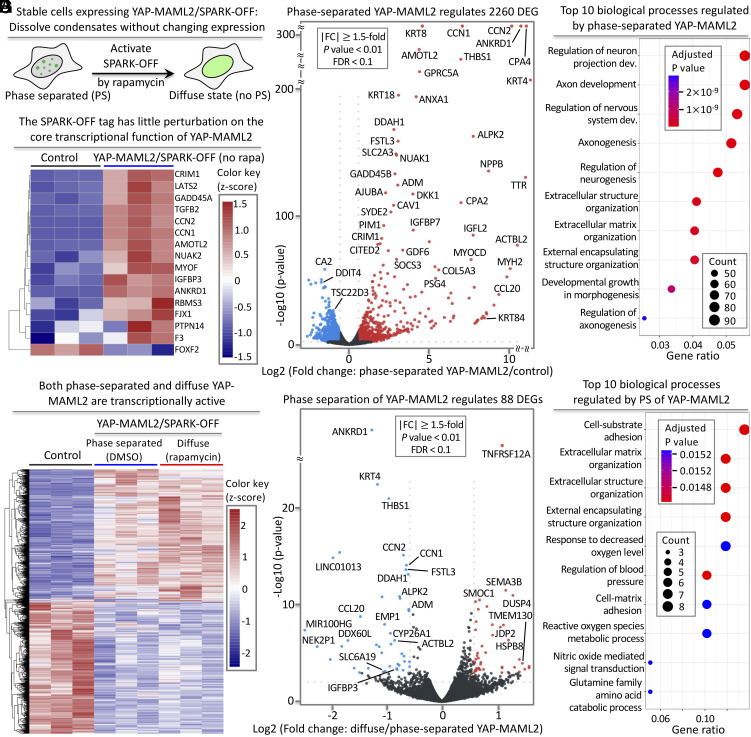
PS of YAP-MAML2 differentially modulates the transcriptome. (*A*) Schematic of SPARK-OFF-driven dissolution of YAP-MAML2 condensates without change of 21 protein level. (*B*) Heatmap of the core target genes of YAP-MAML2. (*C*) Volcano plot showing DEGs that are regulated by YAP-MAML2 condensate. mRNAs showing significant up- and downregulation (|FC| ≥ 1.5, *P*-value < 0.01, FDR < 0.1) are marked in red and blue, respectively. Black dots represent mRNAs with no significant changes. (*D*) GO enrichment analysis of the YAP-MAML2 condensate-regulated biological processes. (*E*) Heat map showing DEGs regulated by YAP-MAML2 in dilute and condensed phases compared to the control cells without expressing YAP-MAML2. The number of the color key represents z-scores. (*F*) Volcano plot showing DEGs that are regulated by PS of YAP-MAML2. mRNAs showing significant up- and downregulation (|FC| ≥ 1.5, *P*-value < 0.01, FDR < 0.1) are marked in red and blue, respectively. (*G*) GO enrichment analysis of the YAP-MAML2 PS-regulated biological processes.

We next tested the effect of YAP-MAML2 condensates (tagged with SPARK-OFF without rapamycin) on the transcriptome (Fig. 7*A*). We processed the engineered cells expressing YAP-MAML2/SPARK-OFF for RNA-seq. First, we tested whether the SPARK-OFF tag itself perturbed the core transcriptional function of YAP-MAML2. We examined 16 core target genes of YAP and found that majority were expressed upon YAP-MAML2/SPARK-OFF expression in HEK293 cells in the absence of rapamycin but these genes were not expressed in control HEK293 cells that do not express YAP-MAML2 (Fig. 7*B*), suggesting little perturbation of the core transcriptional function of YAP-MAML2, and SPARK-OFF is an appropriate tool to use.

Then, we examined the effect of phase-separated YAP-MAML2 on the transcriptome. We calculated differentially expressed genes (DEGs; *P*-value < 0.01, |Log_2_FC| ≥ 0.58, FDR < 0.1). We identified ~2,260 DEGs with ≥1.5-fold change in transcript level, including ~1,270 up-regulated genes and ~990 down-regulated genes (Fig. 7*C* and Dataset S1), consistent with previous reports (2, 60, 61) and also consistent with our imaging data that the YAP-MAML2/SPARK-OFF condensates contain transcriptional machinery (Fig. 6 *B*–*E*). Gene ontology (GO) enrichment analysis reveals that the DEGs are strongly linked to several YAP-related biological processes such as extracellular matrix organization (Fig. 7*D*) (2, 60, 62). Therefore, our results indicate that SPARK-OFF has no or little perturbation on the YAP-MAML2 transcriptional function and that the phase-separated YAP-MAML2 activates known transcriptional programs of YAP. This is consistent with the fact that the TEAD-binding domain of YAP is retained in the fusion protein.

### The Diffuse YAP-MAML2 is Transcriptionally Active and Regulates YAP Target Genes.

We next tested whether the diffuse YAP-MAML2 induced by rapamycin/SPARK-OFF is functional and transcriptionally active in regulating the core YAP-MAML2 target genes and the transcriptome. We characterized transcriptomic changes upon rapamycin treatment of YAP-MAML2/SPARK-OFF expressing cells, in comparison to the control cells without YAP-MAML2. We identified ~2,000 DEGs (*P*-value < 0.01, |Log_2_FC| ≥ 0.58, FDR < 0.1) with ≥1.5-fold change in gene expression, including upregulation of ~990 genes and downregulation of ~1,100 genes (*SI Appendix*, Figs. S8 and S9 and Dataset S2). These include many core YAP target genes including *CTGF* and *CYR61* (2, 61). GO enrichment analysis reveals that the diffuse YAP-MAML2 regulates several known YAP-related biological processes (*SI Appendix*, Fig. S9) (2, 63).

### PS of YAP-MAML2 Selectively Modulates the Transcriptome.

We then asked to what extent transcriptional changes are different between the phase-separated YAP-MAML2 (without rapamycin treatment) and diffuse YAP-MAML2 (with rapamycin treatment and thus no PS), i.e., how much PS contributed to function on top of expression of diffuse YAP-MAML2. Of note, these conditions have the same expression levels, but the former has YAP-MAML2 PS and thus contains YAP-MAML2 condensates while the latter has no YAP-MAML2 PS and thus contains no YAP-MAML2 condensates. We determined DEGs (*P*-value < 0.01, |Log_2_FC| ≥ 0.58, FDR < 0.1) by comparing the RNA-seq data of the engineered cells expressing the YAP-MAML2/SPARK-OFF with versus without rapamycin. We identified 88 DEGs (*P*-value < 0.01, |Log_2_FC| ≥ 0.58, FDR < 0.1) that are regulated by YAP-MAML2 PS with ≥1.5-fold change in transcript levels, including 44 up-regulated genes and 44 down-regulated genes (Fig. 7 *E* and *F*, *SI Appendix*, Fig. S10, and Dataset S3). These genes are therefore regulated differentially by the process of YAP-MAML2 PS. In contrast, none of these 88 DEGs were regulated by rapamycin itself when we compared the RNA-seq data of rapamycin versus DMSO-treated engineered cells expressing the YAP-MAML2/SPARK-OFF control without mCherry (*SI Appendix*, Fig. S11 and Dataset S4). GO enrichment analysis reveals that the YAP-MAML2 PS–regulated genes are strongly linked to multiple YAP-related biological processes including cell-substrate and -matrix adhesions (Fig. 7*G*).

Which genes are affected by PS? Several genes that are up-regulated by YAP-MAML2 expression, for example, *ANKRD1, CTGF, CYR61, THBS1,* and *IGFBP3*, are further up-regulated by PS. This means that 1) *ANKRD1, CTGF, CYR61, THBS1,* and *IGFBP3* are up-regulated by diffuse YAP-MAML2 (compared to the control); 2) and their expression is further increased upon PS of YAP-MAML2 forming condensates. This is also in agreement with our RT-qPCR results for *CTGF* and *CYR61* (Fig. 6*I*). In total, <5% of the up-regulated genes are further enhanced by YAP-MAML2 PS. Our findings indicate that transcription of the most genes is not influenced by YAP-MAML2 PS. In other words, the formation of YAP-MAML2 condensates from the diffusively distributed state does not affect transcript levels for the majority of the genes. Therefore, YAP-MAML2 PS has no function on transcription of these genes, even though the YAP-MAML2 condensates are transcriptionally active. In summary, our data indicate that PS differentially modulates YAP-MAML2-regulated genes.

## Discussion

We have developed and applied a powerful chemogenetic tool, SPARK-OFF, that enables us to dissolve biomolecular condensates without changing expression levels of the driver of PS, here YAP-MAML2 and TAZ. Treatment with rapamycin to activate SPARK-OFF dissolves the condensates of YAP-MAML2 and TAZ on a timescale of 4 min without changing their protein levels in the nucleus. Therefore, SPARK-OFF allows us to compare gene expression between the phase-separated and the non-phase-separated transcription factors and co-activators. This enables us to determine role of PS in transcription.

PS has been increasingly linked to transcription, and growing evidence indicates that transcriptional condensates occur in functional and developmental states (64) as well as in disease (9, 25, 65–67). However, whether PS mediates unique functions in transcription that are not mediated by diffuse complexes has remained controversial. Here, we show that PS influences the expression of only a small fraction of YAP-MAML2-responsive genes, cautioning the assignment of special functions to condensates without evidence. Our results demonstrate the transcriptional activity of YAP-MAML2 condensates; they recruit transcriptional machinery and contain nascent RNA. Our transcriptome analysis further confirmed that both the phase-separated and the non-phase-separated (after dissolution by SPARK-OFF) YAP-MAML2 resulted in the transcriptional regulation of core YAP target genes. However, we also found in our comparison of the phase-separated vs. the diffuse state (i.e., non-phase-separated state) of YAP-MAML2, a unique comparison enabled by SPARK-OFF, that most YAP-MAML2-responsive genes do not undergo expression changes upon dissolution of YAP-MAML2. Only <5% of genes that are promoted by diffuse YAP-MAML2 show further promotion upon PS. Furthermore, many PS-regulated genes are oncogenes including *CYR61*, suggesting a possible correlation between PS and tumorigenesis. However, this does not mean a causal relationship, which requires further investigation. Our results, on the other hand, also emphasize that diffuse complexes formed by YAP-MAML2 with the transcriptional machinery are sufficient for strong transcriptional activity and that PS is a by-product of YAP fusion event resulted from chromosomal translocation rather than a state that alters function broadly.

Our work shows that the transcriptional activity across most genes remains constant upon PS, indicating that the phase-separated YAP-MAML2 represents a state with similar activity as the diffuse state, at the same total protein concentration. The formation of condensates by PS, whether it is due to mutations or gene fusion event or an increase in protein concentration, would concentrate interacting proteins and signaling components and therefore likely result in increased function (15, 17, 18, 20–22, 68). However, our results lead us to predict that the same changes without PS would result in a similar functional increase. Biomolecular condensates can possess emergent properties ranging from activating reactions (69) to filtering noise (70, 71) and changing translation patterns (72) but to elucidate these functions requires careful characterization and dissociation of function (31).

Why is the transcription of a fraction of genes altered upon YAP-MAML2 PS? These effects may be related to emergent properties of condensates such as their ability to coalesce. This is a property not inherent in small diffuse complexes, which have a fixed size distribution at a given concentration and do not grow over time (73). Coalescence of transcriptional condensates can alter chromatin structure and thereby likely bring genes into the vicinity of strong enhancers that alter their expression (25). Such effects may become more dominant the longer the phase-separated state persists, although our data show that YAP-MAML2 condensates disassemble during mitosis and that their lifetimes and their ability to coalesce and ripen are therefore naturally limited.

We conclude that YAP-MAML2 condensates do not mediate a super-proportional fraction of the YAP-MAML2-related activity, which is consistent with another transcription factor N-myc that also shows differential regulation of gene transcription by PS with only a small proportion of regulated genes (74). Nonetheless, condensates may represent emerging interesting drug targets because therapeutics may be enriched within them by direct binding of targets and through binding of other components in condensates and physicochemical effects such as solubility effects (65, 75). While our work addresses the role of PS specifically in YAP-MAML2 transcriptional activity, we expect that many of our findings will hold in other systems and will open new directions for understanding the role of PS in transcription in general.

## Materials and Methods

### mEGFP Concentration and Fluorescence Intensity Standard Curve.

We purified mEGFP protein and estimate the concentration through dividing the absorbance value by extinction co-efficiency. The protein was then serial diluted and imaged under the same Nikon Eclipse Ti inverted microscope and parameters as for YAP-MAML2 PS curve. Fluorescence intensity (counts/pixel) under 488 channel was recorded to plot the mEGFP concentration–fluorescence standard curve (*SI Appendix*, Fig. S1).

### PS Analysis.

HEK293 cells expressing mEGFP-YAP-MAML2 with or without rapamycin were imaged at same parameter. Using the three dimensional (3D) Objects Counter function in ImageJ, a low threshold of fluorescence intensity was set for each cell. The calculated mean fluorescence intensity was used to determine the average YAP-MAML2 protein concentration by comparing with the purified mEGFP concentration vs fluorescence intensity. A higher threshold was set and adjusted for each cell to select the YAP-MAML2 condensates. Total condensate fluorescence 87 intensity in each nucleus was calculated using ImageJ. SPARK value was then determined and plotted to generate the PS curve.

### Western Blot.

Cell lysate was resolved on NuPAGE 4 to 12% Bis-Tris gel, then transferred to nitrocellulose membrane. The membrane was then blocked, incubated with rabbit anti-TAZ polyclonal antibody (Millipore Sigma HPA007415, 1,000×), or rabbit anti-YAP polyclonal antibody (Cell signaling 4912S, 1,000×) and then incubated with an Horseradish peroxidase (HRP)-conjugated anti-rabbit secondary antibody (Cell signaling 7074S, 3,000×). HRP chemiluminescent substrate (Thermo scientific 34580) was added to the membrane, then imaged using Bio-rad Chemidoc XRS system or film (Prometheus 30-507L) exposure. For β-actin, the staining process was similar, but the primary antibody (Santa Cruz sc-47778, 3,000×) and HRP-conjugated anti-mouse secondary antibody (Cell signaling 7076S, 3,000×) were used.

### IF and Nascent-RNA Labelling.

HEK293 cells and ES-2 cells were fixed with 4% paraformaldehyde, permeabilized with 0.5% Triton X-100 in Phosphate-buffered saline (PBST), and blocked with 2% Bovine Serum Albumin (BSA) and 10% goat serum. HEK293 cells were next incubated with anti-RNA polymerase II CTD repeat YSPTSPS (phospho S5) antibody (1:200 dilution, Abcam, ab5408), Med1 antibody (1:2,000 dilution Santa Cruz sc-74475). ES-2 cells were incubated with YAP rabbit polyclonal antibody (1:200 dilution, Cell signaling 4912S). Then, HEK293 cells were incubated with Alexa Fluor 555-conjugated secondary antibodies (1:200 dilution, Abcam, ab150114) and ES-2 cells were incubated with Alexa Fluor 488-conjugated anti-rabbit IgG Fab (1:200 dilution, Cell signaling, 4412S). The nascent RNA was labeled with Click-iT RNA Alexa Fluor 594 imaging kit (Thermo Fisher, C10330) following the manufacturer’s protocol.

### RNA Sequencing and Transcriptome Analysis.

The raw data were processed with fastp. STAR was used to map reads to the human genome (hg38) by default setting. edgeR was applied to the raw counts to identify DEG. Then, Venn diagrams were generated by custom R scripts. Gene heatmap was plotted using DEG by transcript per million (TPM) normalization. GO analysis and Kyoto Encyclopedia of Genes and Genomes analysis were performed with ClusterProfiler (7) each library.

## Supplementary Material

Appendix 01 (PDF)

Dataset S01 (XLSX)

Dataset S02 (XLSX)

Dataset S03 (XLSX)

Dataset S04 (XLSX)

Movie S1. Rapamycin-activable SPARK-OFF dissolves YAP-MAML2 condensates that are labeled by SPARK-OFF, with no perturbation to other nuclear condensates that are not labeled by SPARK-OFF. NPM1 is tagged with a red fluorescent protein mKO3. YAP-MAML2 is labeled by SPARK-OFF. Cells expressing the tagged constructs were treated with rapamycin. The images were taken every 20 seconds. The NPM1 (nucleolus) is shown by red color. YAPMAML2 is shown by green color. Here the non-fluorescent mCherry mutant is used in the SPARK-OFF.

## Data Availability

All data are available in the main text or supporting information.
