# Supplementary material for: Phase separation of YAP-MAML2 differentially regulates the transcriptome
Source: Proc Natl Acad Sci U S A. 2024 Feb 5;121(7):e2310430121. doi: 10.1073/pnas.2310430121 (PMC10873646; doi:10.1073/pnas.2310430121)
Supplement: Supplementary file 1 — Appendix 01 (PDF) [file pnas.2310430121.sapp.pdf]

Supplementary Materials

Materials and Methods

Plasmid construction. Codon optimized FKBP and Frb were gifts from James A. Wells (UCSF). TAZ was amplified from HEK293 cDNA library. SUMO and TEAD4 were amplified from The CCSB Human ORFeome8.1 Collection (donated by Marc Vidal) (1). mCherry-AuroraA-C-7 and sfGFP-N1 were gifts from Michael Davidson (Addgene plasmid # 54997 and #54737). mKO2-N1 was a gift from Michael Davidson & Atsushi Miyawaki (Addgene plasmid # 54625). mKO3 was obtained by introducing the M176F mutation to mKO2 for brighter fluorescence in cells (2, 3). mRuby3 was a gift from Salvatore Chiantia (Addgene plasmid # 127808). mIFP and IFP2 were previously developed by the Shu Lab. pHR_SFFV was a gift from Wendell Lim (Addgene plasmid # 79121). The details of all constructs in this study are listed in Table S1. All plasmid constructs were created by standard restriction enzyme digestion and ligation method and confirmed by exhaustively sequencing the cloned fragments. mCherry, mKO3, mIFP, IFP2, mRuby3, mEGFP, sfGFP, SUMO-mCherry and NLS-mCherry were fused to Frb domain with a flexible linker. pLJM1_mEGFP-YAP-MAML2 was previously generated by the Holland lab. FKBP was fused to its N-terminal with a flexible linker resulting in pLJM1_FKBP-mEGFP-YAP-MAML2. mKO3-TEAD4, CEL-FKBP-eGFP-HOTag and ZIF-EGFP-HOTag6 were previously generated by the Shu lab. HOTag6 in FKBP-EGFP-HOTag6 was replaced with TAZ resulting in FKBP-eGFP-TAZ.

Cell culture and live cell imaging. HEK293 cells were cultured in Dulbecco's Modified Eagle medium (DMEM) high glucose, supplemented with 10% heat inactivated Fetal Bovine Serum (FBS), penicillin (100 units/mL) and streptomycin (100 μg/mL). All culture supplies for HEK293 cells were obtained from Gibco. ES-2 cells were cultured in McCoy's 5A Medium (ATCC 30-2007), supplemented with 10% FBS, penicillin (100 units/mL) and streptomycin (100 μg/mL).

HEK293 cells were transiently transfected with the plasmid using calcium phosphate transfection reagent, or infected with the lentivirus containing the plasmid. Cells were grown in 35 mm glass bottom, four-compartments dishes (Greiner Bio-One 627870). Transfection was performed when cells were cultured to ~50% confluence. Cells were imaged 24 hours after transient transfection. Time-lapse imaging was performed with the aid of an environmental control unit chamber (InVivo Scientific), which was maintained at 37 °C and 5% CO_2.

Confocal microscopy. Samples were imaged on a Nikon Eclipse Ti inverted microscope equipped with a Yokogawa CSU-W1 confocal scanner unit (Andor), a digital CMOS camera ORCA-Flash4.0 (Hamamatsu), a ASI MS-2000 XYZ automated stage (Applied Scientific Instrumentation) and Nikon Plan Apo λ 20X air (N.A. 0.75), Nikon Apo TIRF 60X oil (N.A. 1.49) and CFI Plan Apo λ 100X oil (N.A. 1.45) objectives. Laser inputs were provided by an Integrated Laser Engine (Spectral Applied Research) equipped with laser lines (Coherent) 405 nm for Hoechst imaging, 488 nm for GFP imaging, 561 nm for RFP imaging and 640 nm for IFP imaging. The confocal scanning unit was equipped with the following emission filters: 460/50 nm for Hoechst imaging, 525/50-nm for GFP imaging, 610/60-nm for RFP imaging and 732/68

nm for IFP imaging. Image acquisition was controlled by the NIS-Elements Ar Microscope Imaging Software (Nikon).

mEGFP concentration and fluorescence intensity standard curve. We purified mEGFP protein and measured its 488 nm absorbance using a Thermo scientific nanodrop 2000C to estimate the concentration through dividing the absorbance value by extinction co-efficiency. The protein was then serial diluted between 0.06 μM and 6 μM, added into 8-well chambers and imaged under the same Nikon Eclipse Ti inverted microscope and parameters as for YAP-MAML2 phase separation curve. The laser power was also measured by a Thorlabs PM100D optical power and energy meter to make sure it is stable and the same as for phase separation curve. Fluorescence intensity (counts/pixel) under 488 channel was recorded to plot the mEGFP concentration-fluorescence standard curve (Supporting Fig. S1).

Phase separation analysis. HEK293 cells expressing mEGFP-YAP-MAML2 were imaged under a spinning disc confocal microscope with 60X objective. HEK293 cells expressing YAP-MAML2/SPARK-OFF before rapamycin addition and after 15 min incubation with rapamycin were imaged at same parameter. Each nucleus was sectioned into multiple slices with 0.5 μm interval. Using the 3D Objects Counter function in ImageJ, a low threshold of fluorescence intensity was set for each cell, to select the whole nucleus. The mean green fluorescence intensity in each nucleus was calculated by the ratio of total fluorescence intensity over volume of the nucleus. The calculated mean fluorescence intensity was used to determine the average YAP-MAML2 protein concentration by comparing with the purified mEGFP concentration vs fluorescence intensity. Next, a higher threshold was set and adjusted for each cell to select the YAP-MAML2 condensates. Total condensate fluorescence intensity in each nucleus was calculated using ImageJ. SPARK value was then determined and plotted to generate the phase separation curve.

SPARK-OFF Time-lapse imaging. Various FP-Frb were co-transfected with CEL-FKBP-eGFP-HOTag and ZIF-EGFP-HOTag6 in HEK293 cells. The SparkDrop-FKBP condensates were induced with 1 μM Lenalidomide (Ark Pharma, AK-47482) for 30 mins. To dissolve SparkDrop-FKBP condensates, 30 nM rapamycin (TargetMol, T1537) or 0.1% DMSO was carefully added to the cells in the incubation chamber when the first round of time-lapse imaging was recorded. FKBP-eGFP-TAZ or FKBP-mEGFP-YAP-MAML2 was co-transfected with nls-mCherry-Frb as TAZ/SPARK-OFF or YAP-MAML2/SPARK-OFF. Replace nls-mCherry-Frb with nls-Frb as their controls.

Western blot. HEK293 cells expressing TAZ/SPARK-OFF and YAP-MAML2/SPARK-OFF were grown on 24 well plates, washed once with PBS, lysed with 100 μL lysis buffer (Cell signaling 9803S) at room temperature for 1 minute, mixed with 37.5 μL NuPAGE LDS sample buffer (NP0007) and 15 μL NuPAGE sample reducing agent (NP0009) and incubated at 70ºC for 10 minutes. Protein samples were resolved on NuPAGE 4-12% Bis-Tris gel (NP0336), then transferred to nitrocellulose membrane under 25 V for 45 min in a Bio-rad Trans-Blot Turbo machine. The membrane was then blocked in 5% non-fat milk at room temperature for 1 hour, incubated with rabbit anti-TAZ polyclonal antibody (Millipore Sigma HPA007415, 1000x) or rabbit anti-YAP polyclonal antibody (Cell signaling 4912S, 1000X) at 4ºC over-night, washed 3 times in PBST (0.1% Tween-20 in PBS), each time for 5 minutes. Then incubated with an HRP-

conjugated anti-rabbit secondary antibody (Cell signaling 7074S, 3000X) for 1 hour at room temperature and washed 3 times, each time for 5 minutes. HRP chemiluminescent substrate (Thermo scientific 34580) was added to the membrane, incubated at room temperature for 5 minutes, then imaged using Bio-rad Chemidoc XRS system or film (Prometheus 30-507L) exposure. For β-actin, the staining process was similar, but the primary antibody (Santa Cruz sc-47778, 3000X) and HRP-conjugated anti-mouse secondary antibody (Cell signaling 7076S, 3000X) were incubated for 1 hour at room temperature.

Immunofluorescence and nascent-RNA labelling. HEK293 cells were plated on fibronectin pre-coated 8-well chambered coverglass (Nunc Lab-Tek II, 155409) and were infected with mEGFP-YAP-MAML2, YAP-MAML2/SPARK-OFF, TAZ/SPARK-OFF and their control. ES-2 ovarian cancer cells were plated on 8-well chambered coverglass without any further treatment. After treating HEK293 cells with 0.01% DMSO or 30 nM rapamycin for 2 hrs, HEK293 cells and ES-2 cells were fixed with 4 % paraformaldehyde in PBS, permeabilized with PBST (0.5% Triton X-100 in PBS) and blocked with 2% BSA and 10% goat serum in PBS. HEK293 cells were next incubated with anti-RNA polymerase II CTD repeat YSPTSPS (phospho S5) antibody (1:200 dilution, Abcam, ab5408) in blocking buffer at 4 °C for overnight, Med1 antibody (1:2000 dilution Santa Cruz sc-74475) at room temperature for 3 hrs. ES-2 cells were incubated with YAP rabbit polyclonal antibody (1:200 dilution, Cell signaling 4912S) at 4ºC over-night. After washing three times with PBST, HEK293 cells were incubated with Alexa Fluor 555-conjugated secondary antibodies (1:200 dilution, Abcam, ab150114) and ES-2 cells were incubated with Alexa Fluor 488-conjugated anti-rabbit IgG Fab (1:200 dilution, Cell signaling, 4412S) at room temperature for 1 hr. After washing with PBST three times, 1 μg/mL Hoechst 33342 was added to each well and the cells were imaged after 10 mins. The nascent RNA was labelled with Click-iT RNA Alexa Fluor 594 imaging kit (Thermo Fisher, C10330) following manufacturer's protocol. HEK293 cells expressing mEGFP-YAP-MAML2, YAP-MAML2/SPARK-OFF, TAZ/SPARK-OFF and their control were treated with 0.01% DMSO or 30 nM rapamycin for 1 hrs, then labelled with 1 mM 5-ethynyl uridine for 1 hr. After fixed with 4 % paraformaldehyde in PBS, permeabilized with PBST (0.5% Triton X-100 in PBS), the cells were incubated with Click-iT reaction cocktail at room temperature for 30 mins to visualize 5-ethynyl uridine.

Lentivirus preparation. TAZ/SPARK-OFF, YAP-MAML2/SPARK-OFF lentiviral plasmids were co-transfected with pAX2 and pVSVG at 3:2:1 ratio into HEK293 cells using polyethylenimine (MilliporeSigma 764965). Lentiviruses were harvested after 48 hours.

Estimation of endogenous YAP-MAML2 protein levels.
To estimate the endogenous YAP-MAML2 protein concentration in ES-2 cells, we compared ES-2 cells with the HEK293 cells expressing mEGFP-YAP-MAML2 (HEK293-YAP-MAMP2). (Supporting Fig. S3). First, we conducted western blot as described above to compare the protein levels. $3x10^5$ of HEK293 or ES-2 cells were used for western blot as described above. The band intensity was measured with ImageJ. Second, ES-2 and HEK293-YAP-MAMP2 were grown in glass-bottom 8-well chambers (Thermo Scientific 155411), stained with 1 μg/mL Hoechst 33342 for 10 minutes, and imaged under the confocal microscope for Hoechst fluorescence with the 60X objective. The nucleus was sectioned into multiple slices with a 0.5 μm interval. The nuclear volume was calculated using the 3D Objects Counter function in ImageJ. Third, HEK293-YAP-

MAMP2 cells were imaged under the GFP channel. The GFP fluorescence intensity was used to calculate mEGFP-YAP-MAML2 concentration in the nucleus of the HEK293-YAP-MAMP2 cells, by comparing its fluorescence intensity with that of purified mEGFP fluorescence intensity and concentration (Supporting Fig. S1). The YAP-MAML2 concentration in ES-2 cell was estimated by comparing the western-blot band intensity (4), nuclear volume of ES-2 vs HEK293-YAP-MAMP2 cells, mEGFP-YAP-MAML2 concentration in the nucleus of HEK293-YAP-MAMP2 cells.

RT-qPCR. HEK293 cells were infected with TAZ/SPARK-OFF, YAP-MAML2/SPARK-OFF and their control in presence of 10 μg/mL Polybrene (Sigma-Alderich, H9268). 1 days after infection, the cells with green fluorescence were collected by FACS sorting and seed $1x10^5$ cells per cm^2. 2 days after FACS sorting, the cells were challenged with 1 μM lenalidomide or 0.01% DMSO for 3 hrs. The total RNA was extracted using a Direct-zol RNA MicroPrep (Zymo Reserch, R2060) and converted to complimentary DNA using a SuperScript™ IV Reverse Transcriptase (Invitrogen, 18090010). The RT-qPCR was carried out on a CFX96 Touch Real-Time PCR Detection System using iTaq Universal SYBR Green Supermix (Bio-Rad, 1725121) with a *GAPDH* control. The following primers were used:

	Forward primer	Reverse primer
GAPDH	GTCTCCTCTGACTTCAACAGCG	CTCTTCCTCTTGTGCTCTTGCTG
CTGF	AGGAGTGGGTGTGTGACGA	CCAGGCAGTTGGCTCTAATC
CYR61	CCTCGGCTGGTCAAAGTTAC	TTTCTCGTCAACTCCACCTC

RNA sequencing and transcriptome analysis. HEK293 cells expressing EGFP, YAP-MAML2/SPARK-OFF and its control (no mCherry) were collected by FACS sorting and seed $1x10^5$ cells per cm^2. 2 days after FACS sorting, the cells were challenged with 1 μM lenalidomide or 0.01% DMSO for 3 hrs. The total RNA was extracted using a Quick-RNA Microprep Kit (Zymo Reserch, R1051). The mRNA library construction and Illumina Novaseq sequencing was performed at Novogene Bioinformatics Technology Co., Ltd. 20 millions of 150 bp paired-end reads per sample were generated. After standard illumina Hiseq demultiplexing, raw data were processed with fastp (5) to remove PCR duplications and cut illumina adaptors. STAR (6) was used to map reads to the human genome (hg38) by default setting. Then the raw counts of each gene were obtained. To identify differentially expressed genes (DEGs), edgeR (7) was applied to the raw counts. Log2 Fold Change (log2FC), P value and false discovery rate (FDR) were calculated using TMM normalization. Then volcano plots were introduced to visualize DEGs analysis results, which defines up-regulated genes by $P < 0.01$, $FDR < 0.05$ and $log2FC > 0.58$, and down-regulated genes by $P < 0.01$, $FDR < 0.05$ and $log2FC < -0.58$. Two groups of DEGs were compared and those genes presented in both groups were considered as overlapped genes. Then Venn diagrams were generated by custom R scripts. Gene heatmap was plotted using differentially expressed genes by TPM normalization. Gene Ontology (GO) analysis and Kyoto Encyclopedia of Genes and Genomes (KEGG) analysis were performed with ClusterProfiler (7) each library. Gene heatmap was plotted using differentially expressed genes by TPM normalization. The raw RNA-seq data and processed data file can be retrieved through the Gene Expression Omnibus with the accession number GSE253678 (8).

Image analysis and Colocalization analysis. For analysis of the SPARK signal, images were processed in ImageJ. The sum of droplets pixel fluorescence intensity and the cells pixel

intensity were scored using Analyze Particle function in imageJ. Quantitative data was plotted in EXCEL. We quantified the percentage of condensate colocalization of mEGFP-YAP-MAML2 condensates, TAZ/SPARK-OFF, YAP-MAML2/SPARK-OFF condensates with mKO3-TEAD4, MED1 antibody staining, RNA polymerase II S5P antibody staining, nascent RNA labeling, in single nucleus using spinning disc confocal microscope at 100X. Each nucleus was sectioned into multiple slices with 0.5 µm interval. To calculate condensate number, the images were imported into ImageJ and the condensates were selected by threshold, which were then added to ROI manager. Total number of condensates was summarized from each slice. The overlapped condensates were selected using AND in ROI manager and the number of overlapped condensates was calculated using Analyze particles.

Table S1. List of all constructs.

Vector	Constructs or name of tools	Notes
pLJM1	mEGFP-YAP-MAML2	Lentiviral vector for constitutive expression of genes (CMV promoter)
pLJM1	FKBP-mEGFP-YAP-MAML2	
pcDNA3.1	mKO3-TEAD4	mKO3: a red fluorescent protein (i.e. mKO2/M176F)
pcDNA3.1	CEL-Frb-eGFP-HOTag3	CEL: c-terminal domain of CRBN (109aa)
pcDNA3.1	ZIF-EGFP-HOTag6	ZIF: zinc finger domain 2 of IKZF1 (31aa)
pcDNA3.1	SUMO-mCherry-FKBP	
pcDNA3.1	mCherry-FKBP	
pcDNA3.1	mKO3-FKBP	
pcDNA3.1	mRuby3-FKBP	
pcDNA3.1	mEGFP-FKBP	
pcDNA3.1	sfGFP-FKBP	
pcDNA3.1	mIFP-FKBP	
pcDNA3.1	IFP2-FKBP	
pHR_SFFV	FKBP-EGFP-TAZ	Lentiviral vector for constitutive expression of genes (SFFV promoter)
pHR_SFFV	NLS-mCherry-Frb	NLS: PAAKRVKLD
pHR_SFFV	NLS-Frb	No mCherry control
	SparkDrop-FKBP	Composed of 2 constructs: CEL-FKBP-EGFP-HOTag3 + ZIF-EGFP-HOTag6
	TAZ/SPARK-OFF	Composed of 2 constructs: FKBP-EGFP-TAZ + nls-mCherry-Frb
	YAP-MAML2/SPARK-OFF	Composed of 2 constructs: FKBP-mEGFP-YAP-MAML2+ nls-mCherry-Frb

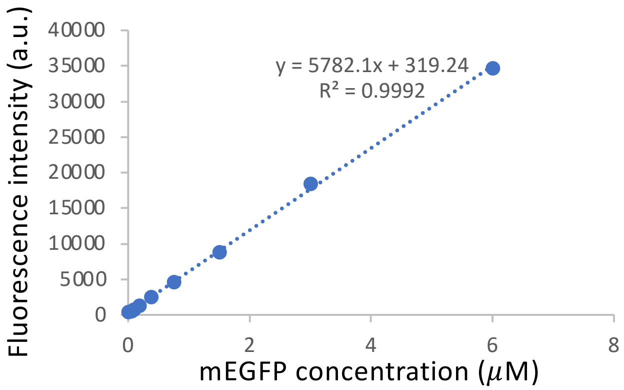

Fig. S1 Relationship of mEGFP fluorescence brightness and concentration. mEGFP was purified and aliquoted at various concentrations. The protein samples were then imaged under the confocal microscope. The fluorescence brightness was recorded, corresponding to mEGFP concentration. The plotted line was used to estimate mEGFP concentration in living cells based on the fluorescence brightness.

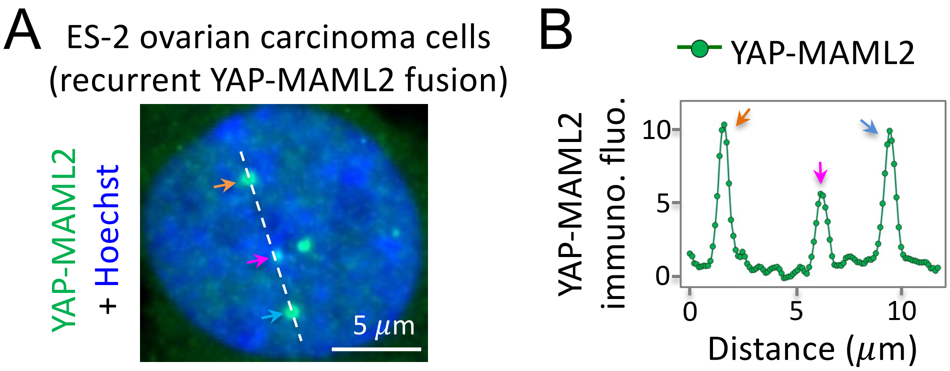

Fig. S2 Immunofluorescence imaging of YAP-MAML2 in ES-2 ovarian cancer cells. (**A**) Fluorescence images of YAP-MAML2 in ES-2 cells. Arrows point to condensates. Cells were stained by antibodies against YAP (green), and Hoechst (blue). (**B**) The fluorescence intensity profile is extracted from the position shown by the dashed line in (A).

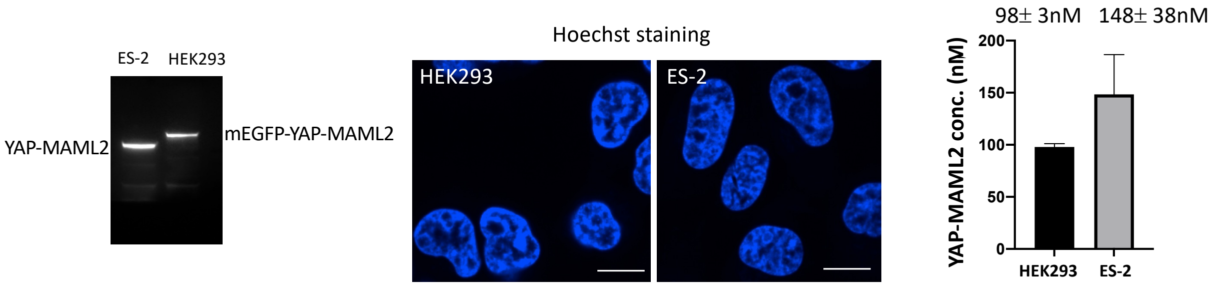

Fig. S3 Estimation of YAP-MAML2 concentration in ES-2 ovarian carcinoma cells. The overall procedure is A) western blot analysis to determine relative amount of YAP-MAML2 in ES-2 (expressing endogenous YAP-MAML2) vs HEK293 cells (expressing exogenous fusion protein). B) Fluorescence imaging (Hoechst)-based measurement of the nuclear volume of ES-2 and HEK293 cells. The mEGFP-YAP-MAML2 concentration in HEK293 cells is determined by comparing the fluorescence intensity of mEGFP-YAP-MAML2 versus that of the purified mEGFP. C) Estimation of the mEGFP-YAP-MAML2 concentration in the nucleus of ES-2 cells. Data are mean ± SD (n = three independent experiments). Scale bar, 10μm.

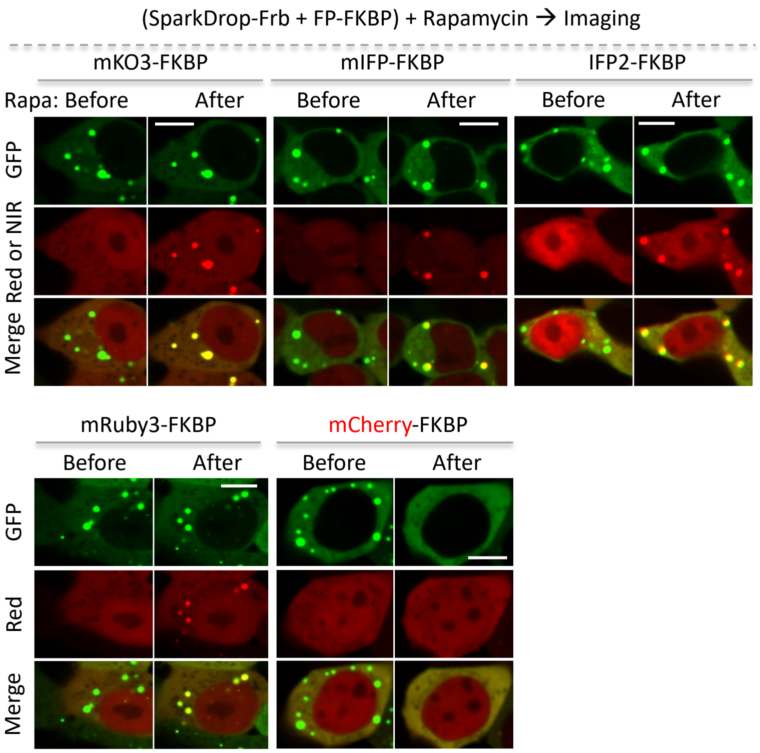

Fig. S4 Multicolor fluorescence images showing recruitment of FP-FKBP to the synthetic condensates that contain Frb upon rapamycin-induced FKBP and Frb interaction. Scale bar: 5 µm.

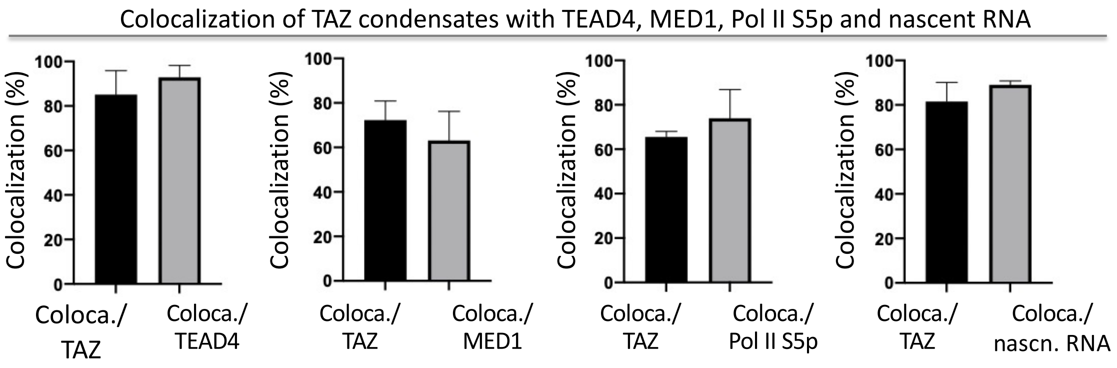

Fig. S5 Percentage of TAZ condensates that colocalize with puncta of other components. The percentage is determined by the ratio of coloca./TAZ = number of colocalized condensates between TAZ and TEAD4 divided by number of TAZ condensates. Equivalent analysis for other pairs is also shown. Data are mean ± SD (n = 13 cells).

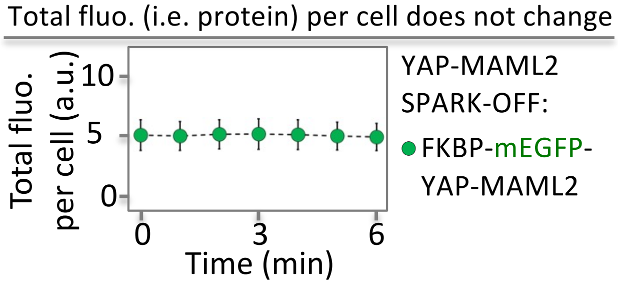

Fig. S6 Quantified total green fluorescence per cell over time upon dissolution of YAP-MAML2 condensates. Data are mean ± SD (n = 3).

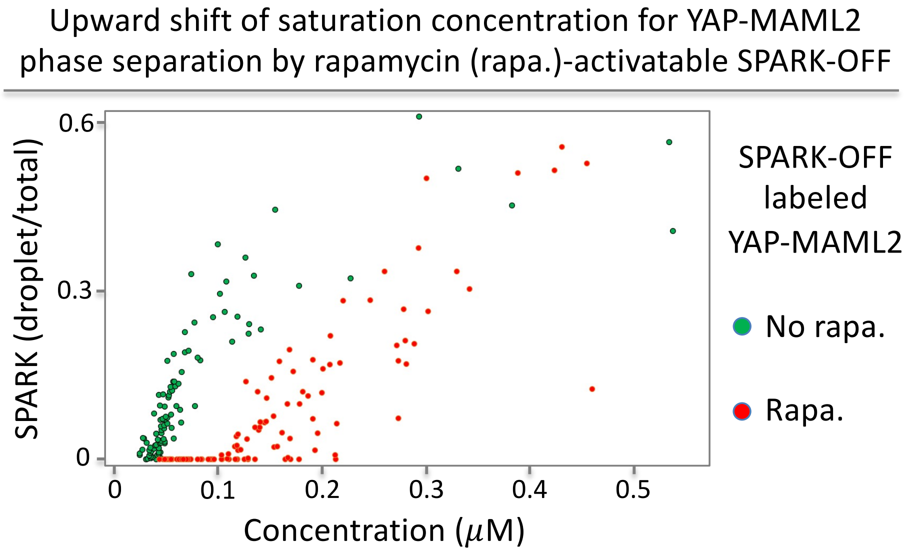

Fig. S7 Shift of saturation concentration of YAP-MAML2 upon rapamycin-activatable SPARK-OFF. SPARK-OFF-tagged YAP-MAML2 was expressed in HEK293 cells. Cells were treated with or without rapamycin. Each green dot represents data from a single cell (102 cells) without treatment of rapamycin. Each red dot represents data from a single cell (114 cells) treated with rapamycin. The concentration was calculated based on the green fluorescence of mEGFP in the labeled YAP-MAML2.

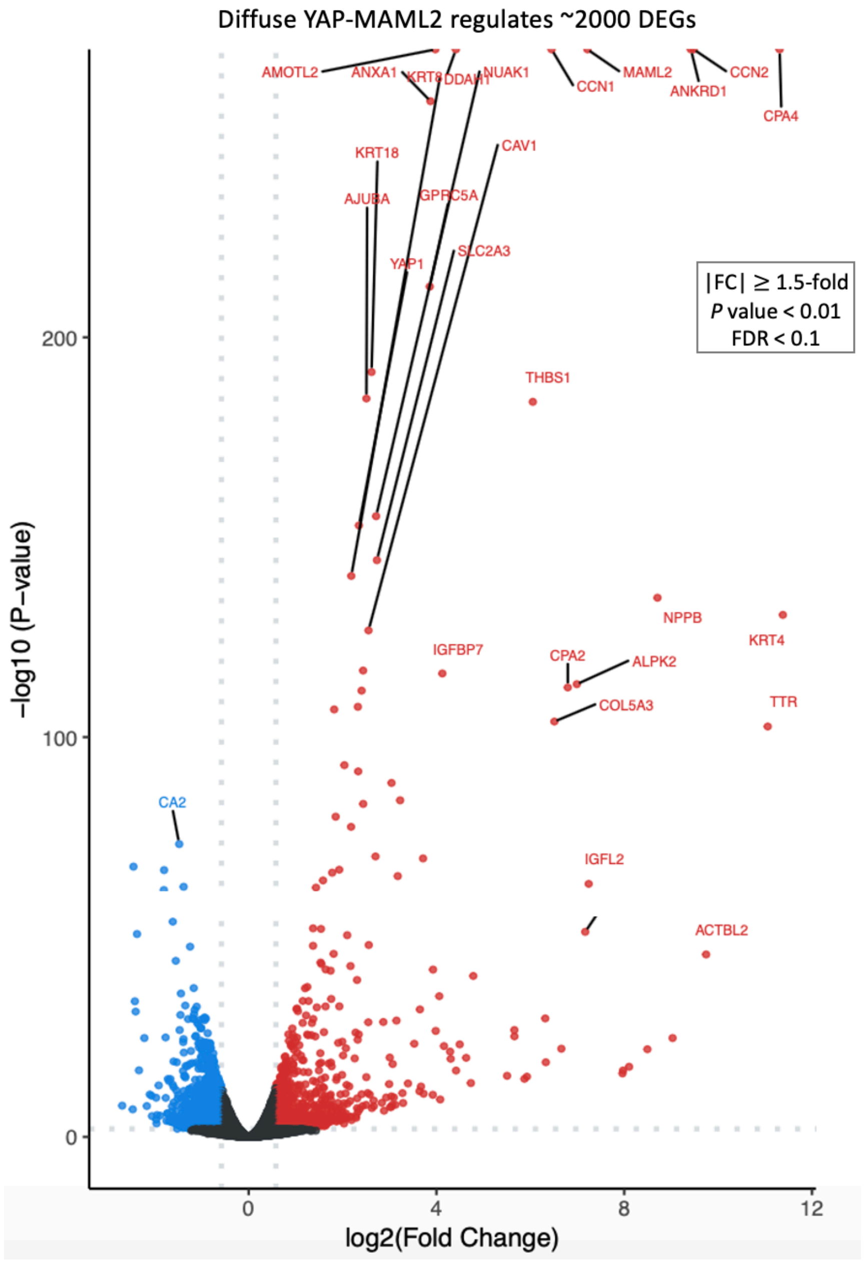

Fig. S8. Volcano plot showing DEGs that are regulated by diffuse YAP-MAML2 (no phase separation). HEK293 cells expressing YAP-MAML2/SPARK-OFF were treated with rapamycin that activates SPARK-OFF. The samples were then processed for RNA sequencing. mRNAs showing significant up- and down-regulation (|FC| ≥ 1.5, p-value < 0.01, FDR < 0.1) are marked in red and blue, respectively. Black dots represent mRNAs with no significant changes.

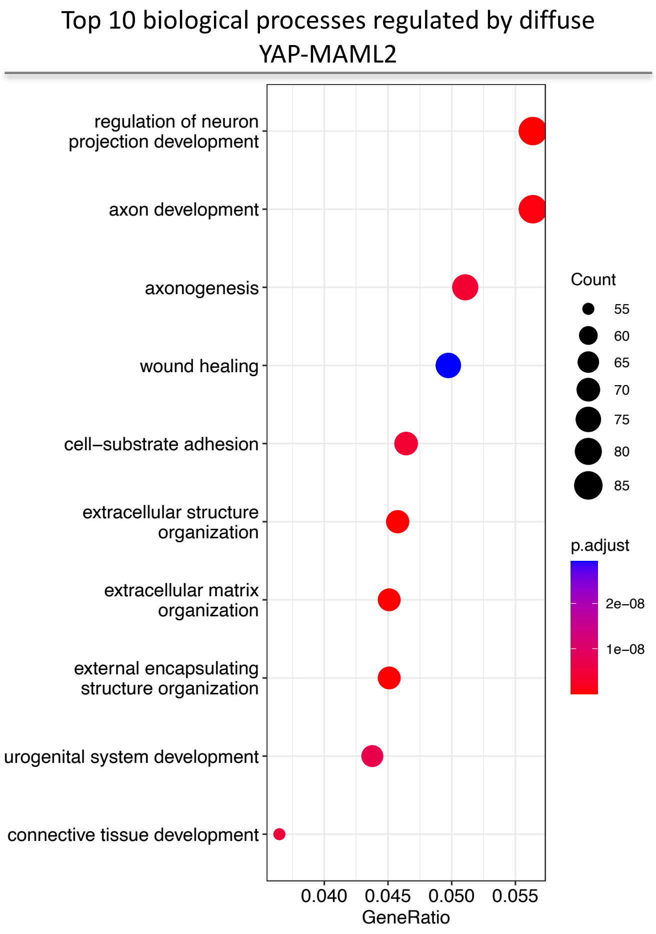

Fig. S9. GO analysis of the top 10 biological processes that are regulated by the diffuse YAP-MAML2 (no phase separation). HEK293 cells expressing YAP-MAML2/SPARK-OFF were treated with rapamycin that activates SPARK-OFF and dissolves YAP-MAML2 condensates. The samples were then processed for RNA sequencing.

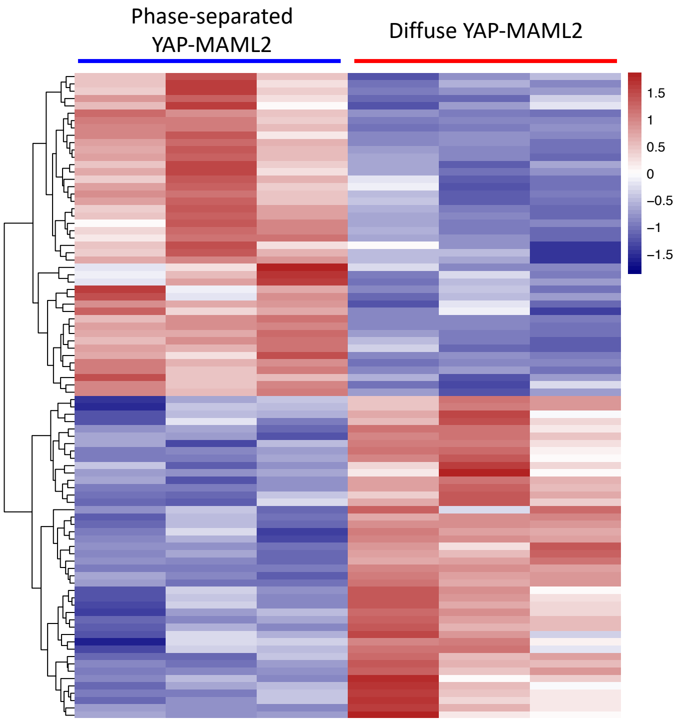

Fig. S10. Heatmap showing up- and down-regulated genes upon YAP-MAML2 condensate dissolution. HEK293 cells expressing YAP-MAML2/SPARK-OFF were treated with or without rapamycin. The samples were then processed for RNA sequencing.

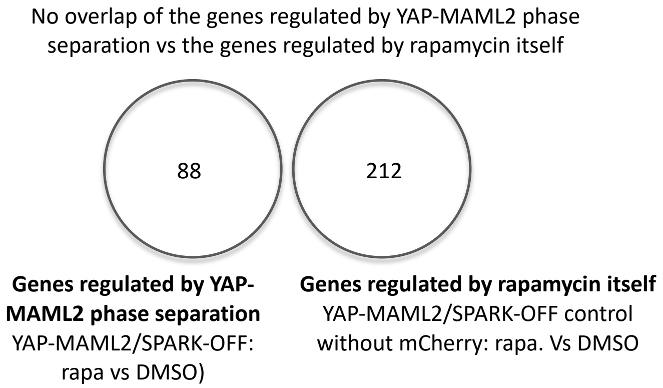

Fig. S11. Rapamycin itself does not affect transcription of the genes that are regulated by YAP-MAML2 phase separation. Venn diagram shows no overlap between the genes regulated by YAP-MAML2 phase separation and those genes regulated by rapamycin itself. The genes regulated by rapamycin itself were determined using the cells expressing YAP-MAML2/SPARK-OFF control without mCherry, treated with or without rapamycin.

Movie S1. Rapamycin-activable SPARK-OFF dissolves YAP-MAML2 condensates that are labeled by SPARK-OFF, with no perturbation to other nuclear condensates that are not labeled by SPARK-OFF. NPM1 is tagged with a red fluorescent protein mKO3. YAP-MAML2 is labeled by SPARK-OFF. Cells expressing the tagged constructs were treated with rapamycin. The images were taken every 20 seconds. The NPM1 (nucleolus) is shown by red color. YAP-MAML2 is shown by green color. Here the non-fluorescent mCherry mutant is used in the SPARK-OFF.

Reference:

1. Wiemann S, et al. (2016) CORRESPONDENCEThe ORFeome Collaboration: a genome-scale human ORF-clone resource. *Nat Methods* 13(3):191–192.

2. Mastop M, et al. (2017) Characterization of a spectrally diverse set of fluorescent proteins as FRET acceptors for mTurquoise2. *Sci Rep*:1–18.

3. Tsutsui H, Karasawa S, Okamura Y, Miyawaki A (2008) Improving membrane voltage measurements using FRET with new fluorescent proteins. *Nat Methods* 5(8):683–685.

4. Pillai-Kastoori L, Schutz-Geschwender AR, Harford JA (2020) A systematic approach to quantitative Western blot analysis. *Analytical Biochemistry* 593:113608.

5. Chen S, Zhou Y, Chen Y, Gu J (2018) fastp: an ultra-fast all-in-one FASTQ preprocessor. *Bioinformatics* 34(17):i884–i890.

6. Dobin A, et al. (2013) STAR: ultrafast universal RNA-seq aligner. *Bioinformatics* 29(1):15–21.

7. Robinson MD, McCarthy DJ, Smyth GK (2010) edgeR: a Bioconductor package for differential expression analysis of digital gene expression data. *Bioinformatics* 26(1):139–140.

8. C.-I. Chung, J. Yang, X. Yang, H. Liu, Z. Ma, F.Szulzewsky, E. C. Holland, Y. Shen, X. Shu, From “Phase separation of YAP-MAML2 differentially regulates the transcriptome.” Available at https://www.ncbi.nlm.nih.gov/geo/query/acc.cgi?acc=GSE253678. Gene Expression Omnibus. Released Jan 23, 2024.
